# Enzymatic cottonseed protein alleviates DSS-induced enteritis in juvenile yellow catfish (*Pelteobagrus fulvidraco*): focus on macrophage polarization and necroptosis in the intestine

**DOI:** 10.1186/s40104-025-01248-z

**Published:** 2025-08-26

**Authors:** Guangju Zhang, Xiaoqiu Zhou, Weidan Jiang, Pei Wu, Yang Liu, Yaobin Ma, Hongmei Ren, Xiaowan Jin, Weiwei Xiao, Ya Li, Lin Feng

**Affiliations:** 1 Animal Nutrition Institute, Sichuan Agricultural University, Chengdu, 611130 China; 2 Fish Nutrition and Safety Production University Key Laboratory of Sichuan Province, Sichuan Agricultural University, Chengdu, 611130 China; 3 Key Laboratory of Animal Disease-Resistance Nutrition, Ministry of Education, Ministry of Agriculture and Rural Affairs, Key Laboratory of Sichuan Province, Sichuan, 611130 China; 4 Chengdu Mytech Biotech Co., Ltd., Chengdu, 610222 Sichuan China

**Keywords:** Dextran sulfate sodium, Enzymatic cottonseed protein, Macrophage polarization, Necroptosis, *Pelteobagrus fulvidraco*

## Abstract

**Background:**

In intensive aquaculture systems, the frequent incidence of enteritis reduces production efficiency and results in significant economic losses. Protein feeds account for 40%–60% of aquafeed expenses, and with the growth of intensive aquaculture, demand for fishmeal as a key protein source outstrips supply, driving up prices. This study investigated the therapeutic potential of reducing dietary protein levels by 3% and adding enzymatic cottonseed protein (ECP) in juvenile yellow catfish with dextran sulfate sodium (DSS)-induced enteritis.

**Methods:**

A total of 1,260 healthy juvenile yellow catfish (*Pelteobagrus fulvidraco*), with an average body weight of 5.90 ± 0.05 g, were randomly allocated into 7 experimental groups, each with 3 replicates. The fish were fed one of seven diets for 10 weeks: a normal-protein diet (42%; NP) and 6 low-protein diets (39%; LP) supplemented with graded levels of ECP at 0% (ECP0), 1% (ECP1), 2% (ECP2), 3% (ECP3), 4% (ECP4), and 5% (ECP5), respectively. Subsequently, 48 fish from each group were selected to receive 1 mL of 6% DSS solution.

**Results:**

Our findings demonstrated that: (1) The DSS + ECP0 group aggravated DSS-induced enteritis in juvenile yellow catfish compared to the DSS + NP group. (2) Dietary supplementation of ECP in LP diets significantly enhanced the enzymatic activity and levels of immunoreactive substances, including LZM, C3, C4, and ACP (*P* < 0.05). Mechanistically, first, ECP supplementation modulated macrophage polarization by inhibiting the M1 phenotype while promoting the M2 phenotype, potentially through the JAK-STAT signaling pathway; second, dietary ECP suppressed the phosphorylation cascade of key necroptosis-related proteins, including RIP1, RIP3, and MLKL, potentially via the NF-κB and MAPK signaling pathways. (3) The DSS + ECP2 group demonstrated comparable or superior efficacy to the DSS + NP group in mitigating DSS-induced intestinal enteritis.

**Conclusions:**

Our results demonstrated that ECP can alleviate DSS-induced enteritis by regulating macrophage polarization and reducing necroptosis. Furthermore, ECP supplementation effectively counteracted the exacerbation of enteritis caused by dietary protein reduction. These findings highlighted the effectiveness and feasibility of ECP in alleviating enteritis and saving protein.

**Graphical Abstract:**

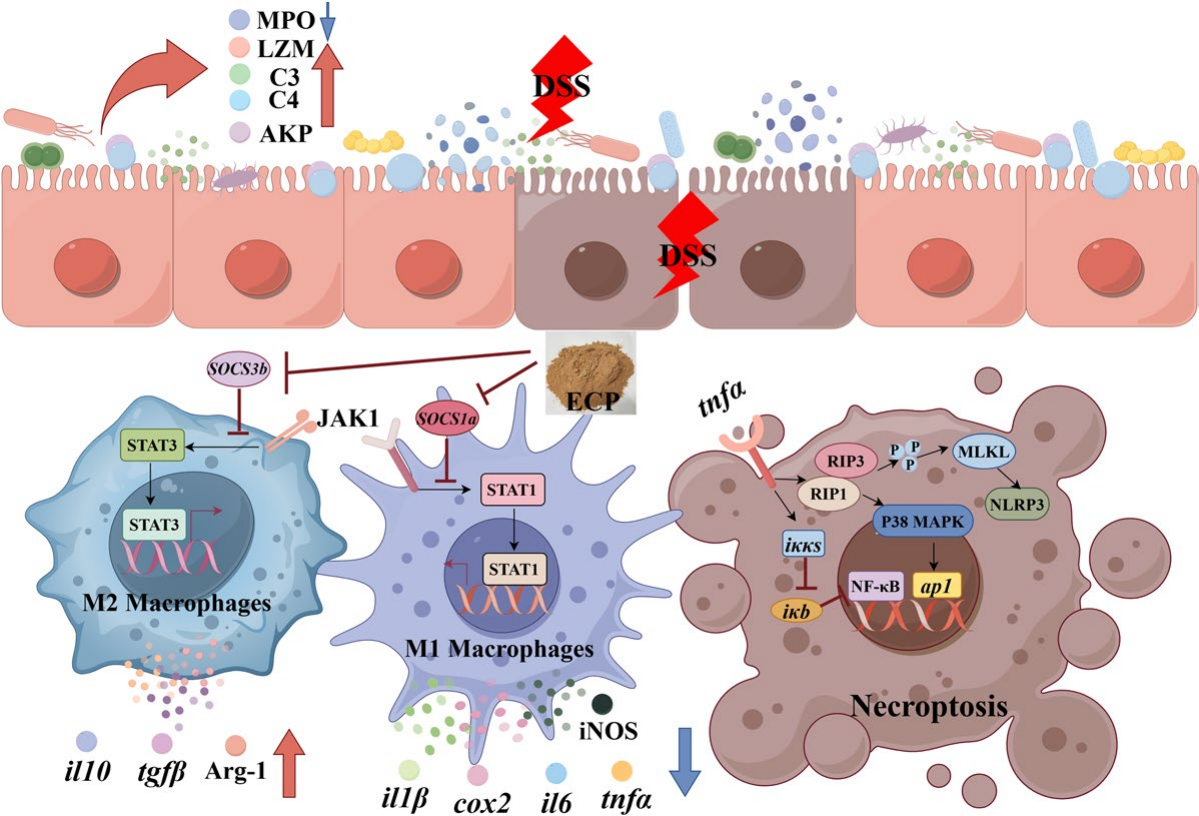

**Supplementary Information:**

The online version contains supplementary material available at 10.1186/s40104-025-01248-z.

## Introduction

Aquatic foods play a significant role in providing high-quality proteins, constituting 15% and 6% of the total global human intake of animal protein and total protein, and aquaculture is expanding as human demand for aquatic products increases [1]. Within antibiotic-free aquaculture systems, fish are susceptible to factors that can lead to enteritis, including anti-nutritional factors in feed [2], suboptimal water quality parameters [3], and pathogenic bacterial challenges [4]. This rise in enteritis cases has had substantial economic implications for the aquaculture industry [5]. The intestine represents a crucial and versatile organ that directly interacts with ingested substances, contributing to digestive and absorptive processes while also maintaining immunological homeostasis through mucosal barriers and resident immune cells [6]. Therefore, identifying effective nutritional strategies to mitigate inflammatory responses in fish has become essential. Enzymatic cottonseed protein (ECP) is a functional peptide product derived from a specialized mixture liquid enzymatic extraction process from cottonseed protein [7]. Our previous study has demonstrated that ECP enhanced fish growth performance, digestive and absorptive capacity, and intestinal structural integrity [8]. However, the effects of ECP on intestinal immune function remain unexplored.

Macrophages are becoming recognized as essential sentinels of the intestinal immune system and necessary maintainers of intestinal homeostasis [9]. Activated macrophages are classified into two main phenotypes: M1 and M2 [10]. While M2 macrophages are mostly linked to anti-inflammatory processes, M1 macrophages are primarily implicated in pro-inflammatory responses [11]. Consequently, the polarization state of macrophages may be a critical determinant in defining the resolution or progression of inflammation and related diseases [12]. Currently, there was no published research investigating the impact of ECP on macrophage polarization. It has been demonstrated that dietary ECP can raise the interleukin 10 (*il10*) and reduce tumor necrosis factor-alpha (*tnfα*) and interleukin 1beta (*il1β*) mRNA expression in the Chinese soft-shelled turtle (*Pelodiscus sinensis*) intestine [13]. Notably, M1 macrophages produce pro-inflammatory factors such as interleukin-6 (IL-6), TNF-α, and IL-1β, whereas M2 macrophages generate anti-inflammatory factors, including IL-10 and transforming growth factor-beta (TGF-β) [14]. These findings suggest that ECP might alleviate intestinal inflammation by modulating macrophage polarization; however, further research is warranted to elucidate the specific pathways involved.

An innate immune response may be initiated by necroptosis, a regulated form of necrosis characterized by the rupture of dead cells and the subsequent release of intracellular components [15]. The molecular cascade of necroptosis is predominantly mediated through the sequential phosphorylation of three pivotal regulatory kinases: receptor-interacting protein 1 (RIP1), receptor-interacting protein 3 (RIP3), and mixed lineage kinase domain-like protein (MLKL), which together form the core regulatory axis of this programmed cell death pathway [16]. Although it has been uncertain whether ECP can mitigate inflammation by inhibiting necroptosis, ECP has been observed to reduce *tnfα* mRNA levels in the liver of largemouth bass (*Micropterus salmoides*) [17]. TNF-α, a common necrosis trigger, facilitates the recruitment of RIP1 and RIP3, as well as the phosphorylation of MLKL, which leads to MLKL oligomerization and its translocation to the plasma membrane, ultimately resulting in necroptosis [18]. Based on these observations, we hypothesized that ECP supplementation may attenuate necroptosis; however, this mechanistic hypothesis necessitates further experimental validation.

The yellow catfish (*Pelteobagrus fulvidraco*) is a species of considerable commercial significance in southern China [19]. As a carnivorous organism, it necessitates a diet with a crude protein content exceeding 40% [20]. Protein is a major contributor to feed costs and plays a pivotal role in the growth performance of aquatic species [21]. In modern aquaculture practices, the strategic incorporation of nutritional supplements to enhance dietary protein utilization is increasingly vital for minimizing production expenses [22]. Our previous research has indicated that the inclusion of ECP following a 3% reduction in dietary protein levels can exert protein-sparing effects by improving digestive efficiency and nutrient absorption [8]. However, whether ECP similarly optimizes protein allocation for immune responses, thereby potentially reducing dietary protein requirements for disease resistance, warrants systematic investigation.

The dextran sodium sulfate (DSS) model has been widely established for investigating intestinal inflammatory responses and immune function in animal studies [23, 24]. This model was primarily characterized by immune cell infiltration [25] and epithelial cell damage [26]. Based on these pathological characteristics, we employed the DSS-induced enteritis model to assess the potential protective effects of ECP addition to diets with a 3% reduction in protein level on juvenile yellow catfish. Our investigation specifically aimed to elucidate two critical molecular mechanisms: macrophage polarization and necroptosis. It was hoped that the results of this study would provide fresh theoretical foundations for understanding the dual properties of ECP, such as its anti-inflammatory and protein-sparing properties.

## Materials and methods

### Diets and experimental management

The composition of the diet is comprehensively outlined in Table S1. The primary protein sources included fish meal, chicken meal, soybean meal, and corn protein powder, while soybean oil and krill oil served as the principal sources of fat. ECP was procured from Mytech Biotech Co., Ltd. (Chengdu, China). The peptide profile of ECP is detailed in Table S2. The cottonseed protein, which contains approximately 65% protein, underwent enzymatic hydrolysis utilizing a combination of protease and cellulase. This reaction was conducted under optimal temperature conditions for 12 h. Upon completion of the hydrolysis process, the product was dried and ground to produce the final ECP preparation. The normal-protein (NP) diet comprised 42% protein. A low-protein (LP) diet, containing 39% protein, was formulated based on previous research indicating the feasibility of a 3% reduction in dietary protein. To ensure a consistent protein level and amino acid balance, varying proportions of ECP were incorporated, while the fishmeal content in the diet was reduced. In the LP diet, ECP was added at different levels: 0% (ECP0), 1% (ECP1), 2% (ECP2), 3% (ECP3), 4% (ECP4), and 5% (ECP5). The prepared diets were stored at 4 °C before use.

Healthy juvenile yellow catfish were initially obtained from a nearby fish farm in Sichuan, China, and were fed a commercial diet for a month to ensure their acclimatization. Following this period, 1,260 juvenile yellow catfish (5.90 ± 0.05 g) were randomly distributed among 21 nets (comprising 7 treatments with 3 nets per treatment, each containing 60 fish). For 10 weeks, the fish were fed to satiation at 5:00, 10:00, 18:00, and 22:00 daily. Any uneaten feed after 60 min was removed. During the entire experimental period, key water quality parameters, including temperature, dissolved oxygen (DO), pH, nitrite concentration, and ammonia levels, were meticulously monitored and recorded as 28.7 ± 1.7 °C, > 6 mg/L, 7.0–8.5, < 0.05 mg/L, and < 0.1 mg/L, respectively.

### Challenge trial

The DSS-induced enteritis model has been successfully established and preliminarily validated in both zebrafish [27] and Orange-spotted grouper (*Epinephelus coioides*) [23]. DSS challenge test has followed the protocol established by Chen et al. [28]. For the preliminary challenge test, 60 healthy juvenile yellow catfish of comparable average weight were randomly allocated into 6 experimental groups (*n* = 10). Initially, a specific ratio of PBS to DSS was used to prepare three DSS solutions at concentrations of 0%, 3%, and 6%. Subsequently, each concentration of DSS solution was rectally administered for 24 h at two different doses of 0.5 mL and 1.0 mL into the intestines of each group of juvenile yellow catfish. Before administering the DSS solution, the feces were gently extruded from the distal intestine. A flat-head needle was inserted approximately 2 cm into the anal opening for solution delivery, followed by maintaining the fish in a head-down position for 10 s to prevent solution leakage. The establishment of the enteritis model was deemed successful with an enteritis incidence exceeding 15% and a mortality rate surpassing 10%, ensuring both pathological relevance and experimental reproducibility. Following the assessment of enteritis incidence and mortality rates, the optimal challenge dose was determined to be 1 mL of 6% DSS solution for subsequent formal experiments. In the formal challenge test, 48 juvenile yellow catfish from each treatment group, matching the group’s average weight, were selected post the growth trial. Experimental groups received 1 mL of 6% DSS solution, while the control group was administered 1 mL of PBS solution, following the same administration protocol outlined for the preliminary challenge test.

### Sample collection

After the challenge was over, the fish was dissected, and the whole intestine was carefully isolated. The part of the mid-intestinal samples was treated in three sections to satisfy various analytical requirements. The first part was thoroughly rinsed with saline and fixed using 4% paraformaldehyde; the second was fixed using 2.5% glutaraldehyde; and the final part was quickly frozen with liquid nitrogen and stored at −80 °C.

### Enteritis morbidity

The severity of intestinal inflammation was quantitatively evaluated using a systematic scoring system based on the observed erythema and edema levels in the proximal, middle, and distal intestinal segments. The scoring criteria were defined as follows: 0 points for intact intestinal morphology; 0–1 point for localized congestion and swelling affecting less than 1/5 of the intestinal segment; 1–2 points for 1/5–1/4 of the segment being involved; 2–3 points for involvement of 1/4 to 1/3 of the segment; 3–4 points for 1/3 to 1/2 segmental involvement; and 4–5 points for extensive inflammation affecting over half of the segment. The enteritis morbidity was calculated by applying the formula: (average total score for each intestinal segment/5) × 100, providing a standardized metric for comparative analysis of inflammatory responses across experimental groups.

### Hematoxylin and eosin (H&E)

Each experimental group collected mid-intestinal tissues from 3 fish, which were fixed in 4% paraformaldehyde and subjected to a series of dehydration steps with increasing ethanol concentrations. Subsequently, the tissues were clarified with xylene and embedded in wax at 58–60 °C. The wax-embedded blocks were then sectioned into 5 µm slices using a microtome. Histological analysis was facilitated by applying H&E staining to these sections. The tissue damage and inflammatory infiltration degree were assessed using an optical microscope (Nikon TS100, Tokyo, Japan).

### Transmission electron microscopy (TEM)

Following treatment with 2.5% glutaraldehyde, mid-intestinal samples from 3 fish in each experimental group were rinsed with a phosphate buffer. Subsequently, the samples were further fixed in 1% osmium tetroxide for 2 h. Dehydration proceeded gradually, starting with 30% ethanol and progressing to 100% ethanol, followed by two additional dehydration steps using 100% acetone. The samples were permeabilized and embedded using acetone and Epon812 embedding agents. Employing an ultrathin sectioning machine (EMUC7, Leica, Wetzlar, Germany), the embedded samples were cut into 80 nm thick ultrathin sections and placed on 200-mesh copper grids. The grids were then stained with uranyl acetate and lead citrate before being air-dried overnight at around 20 °C. Finally, images of the prepared copper grids were taken and examined using a JEM 1400 FLASH (JEOL, Japan) transmission electron microscope.

### Biochemical indicators

An appropriate number of mid-intestinal samples from 6 fish in each experimental group were weighed to prepare a 10% tissue homogenate for the measurement. A corresponding volume of saline solution (w:v = 1:9) was then added to the samples. After using a tissue grinder to homogenize the mixture, the supernatant was extracted by centrifuging it for 10 min at 4,000 r/min. The levels of lysozyme (LZM) (A050-1-1), myeloperoxidase (MPO) (A044-1-1), acid phosphatase (ACP) (A060-1-1), and inducible nitric oxide synthase (iNOS) (A014-1-2) were assessed using commercially available kits from the Bioengineering Institute in Nanjing, China. Similarly, commercial kits from ERKN Biotechnology (Zhejiang, China), were used to measure the concentrations of complement 3 (C3) (231003) and complement 4 (C4) (230502). A commercial kit (YJ650874, Mlbio Biology, Shanghai, China) was used to determine the arginase 1 (Arg-1).

### Real-time fluorescence quantification (q-PCR)

The mid-intestinal tissues of 6 fish from each experimental group were selected for q-PCR analysis. Intestinal RNA was first extracted using Trizol lysate (Vazyme Biotech Co., Ltd., Nanjing, China), and agarose gel electrophoresis (1%) and spectrophotometric (A_260_/A_280_) analyses were used to confirm RNA quality and purity, respectively. Subsequently, reverse transcription of the RNA was conducted using the Prime Script RT kit (Takara Bio, Kusatsu, Japan). For normalization purposes, β-actin was selected as the reference gene based on in-group screening. Quantitative PCR analysis was performed using a 20 μL reaction system containing 2 × SYBR Green qPCR Mix (Aidlab Biotechnologies Co., Ltd., Beijing, China). Amplification and real-time fluorescence detection were conducted using the QuantStudio5 Real-Time PCR System (Thermo Fisher Scientific, Waltham, MA, USA). The comparative threshold cycle (2^−ΔΔCT^) approach was used to quantify gene expression after normalizing to reference genes. Table S3 provides a detailed description of the precise primer sequences used for target gene amplification.

### Western blotting (WB)

For WB analysis, mid-intestinal tissues of 6 fish from each experimental group were chosen. The intestinal samples stored at −80 °C were combined with 800 μL of RIPA/PMSF (80:1) lysate, ground at low temperature, and the supernatant was collected. A BCA kit was used to measure the protein concentration (Beyotime, China). To ensure constant protein levels, the supernatant was mixed with the corresponding 5 × sampling buffer and stored at −80 °C after being denatured for 10 min at 95 °C, and then preserved for later use. SDS-PAGE electrophoresis was used to separate the target proteins at room temperature, after which they were subsequently wet-transferred to a PVDF membrane. At room temperature, a protein-free quick closure solution (Solarbio, China) sealed the PVDF membrane for 10 min. The membrane was treated for 17 h at 4 °C with the appropriate primary antibody to ensure specific binding. The membrane was thoroughly washed with TBST before being incubated with a secondary antibody for 1 h. The final step involved color development using an ECL kit (Beyotime, China) under light protection. The developed membrane was imaged with the ChemiDoc imaging system (Bio-Rad, USA), and the protein bands were quantitatively analyzed in grayscale using ImageJ software. Detailed information about the primary antibodies used is provided in Table S4.

### Immunofluorescence (IF)

 The mid-intestinal tissue sections of 3 fish were selected for the IF observation from each experimental group. The prepared sections were deparaffinized and rehydrated, inactivated, and antigenically repaired. The sections were washed with PBS solution, followed by a dropwise addition of standard goat serum sealing solution (5% BSA) for 1 h. Then, drop the primary antibody (1:100) and incubate the slides in a wet box at 4 °C for 17 h. After treating the sections for 1 h at room temperature with Alexa Fluor 488-labeled goat anti-rabbit IgG (Beyotime, China), they were rinsed with PBS solution. An anti-fluorescence quencher (with DAPI) (Solarbio, China) was then applied, and coverslips were finally placed on top. Placed under an inverted fluorescence microscope (Lecia DMI14000B) to take pictures and quantify using Image J.

### Calculation and statistical analyses

Date are presented as the mean ± standard deviation. Before conducting independent samples *t*-test and one-way analysis of variance (ANOVA), the data were assessed for normality using the Shapiro-Wilk test (*n* < 50). *P* > 0.05 indicated that the sample was drawn from a normally distributed population. For datasets violating the normality assumption, a logarithmic transformation was applied to achieve normal distribution. Subsequently, homogeneity of variance was evaluated using Levene’s test. *P* > 0.05 indicated equal variances across groups, thereby satisfying the assumptions for one-way ANOVA and independent samples *t*-tests. Games-Howell post hoc tests were applied for multiple comparisons when unequal variances were detected (*P* < 0.05 for heterogeneity tests). A one-way ANOVA model in SPSS 22.0 was used to statistically analyze the data from the LP diet groups (DSS + ECP0 to DSS + ECP5). The CONTRAST program in SPSS 22.0 assessed the linear and quadratic effects of increased ECP in LP diets. The Duncan method was employed for multiple comparisons of treatment mean values, with a significance level of *P* < 0.05. Data from the DSS + NP group were compared with those from the PBS, DSS + ECP0, and DSS + ECP2 groups using independent samples *t*-test in SPSS 22.0. The *t*-distribution was used to assess differences, with *P* < 0.05 indicating significance.

## Results

### Effects of 3% reduction in dietary protein level on DSS-induced enteritis in juvenile yellow catfish

#### Enteritis morbidity, mortality rate, and intestinal organization

**Fig. 1:**
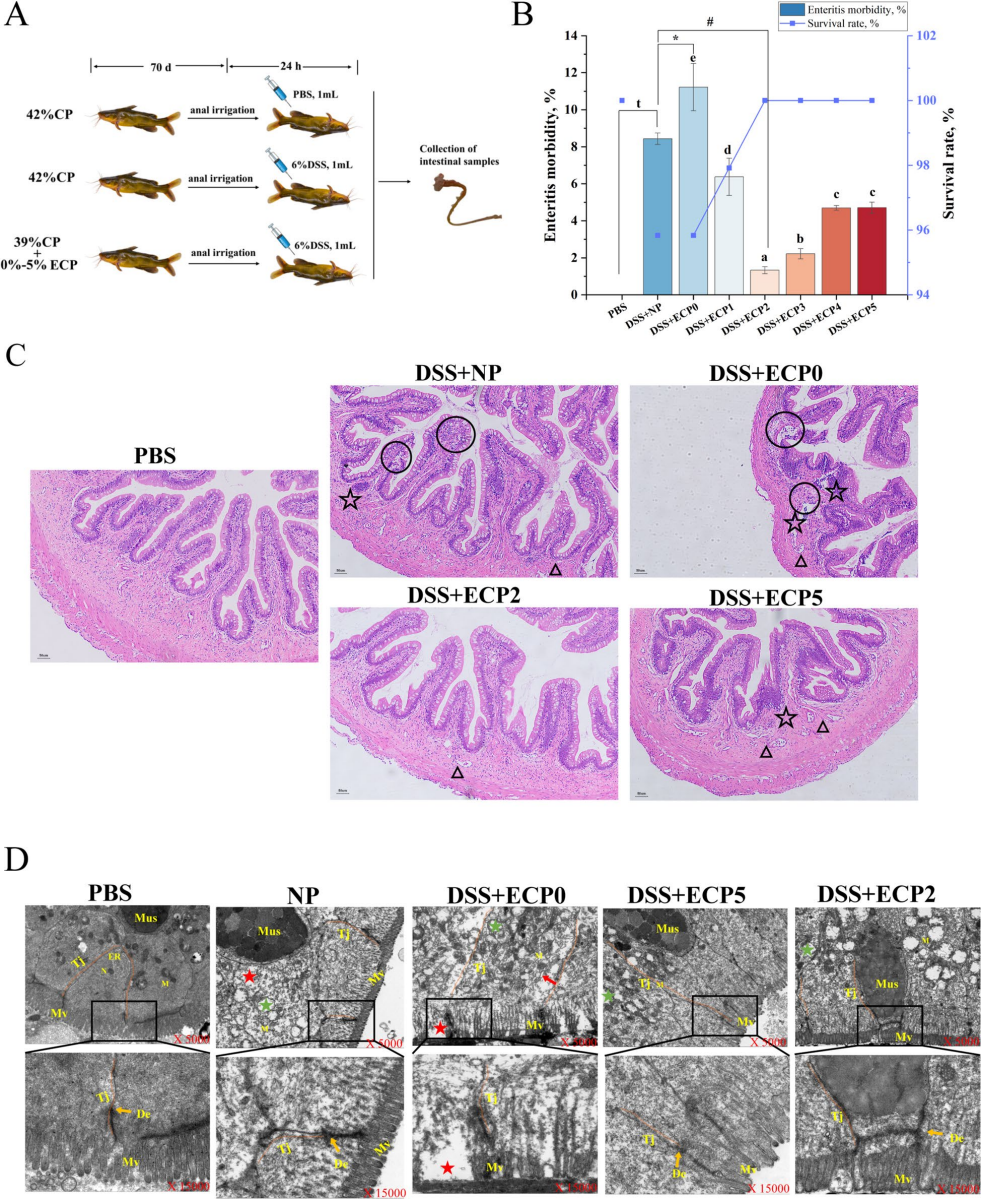
Effects of different levels of ECP on survival rate, enteritis morbidity, and intestinal tissue structure in juvenile yellow catfish following DSS-induced enteritis. **A** Schematic diagram of the experimental design. **B** survival rate and enteritis morbidity, *n* = 48. One-way ANOVA was used for data from the DSS + ECP0–5 groups, and mean values with different superscripts were significantly different (*P* < 0.05). Data from the DSS + NP group were compared with those from the PBS, DSS + ECP0, and DSS + ECP2 groups using independent samples *t*-test. ^t^Indicates a significant difference between the PBS group and DSS + NP group (*P* < 0.05); ^*^Indicates a significant difference between the DSS + NP group and ECP0 group (*P* < 0.05); ^#^Indicates a significant difference between the DSS + NP group and DSS + ECP2 group (*P* < 0.05). **C** Hematoxylin–eosin (HE) staining results (magnification 200 ×, scale bar = 50 μm), circle, vacuolar degeneration; triangle, hyperemia; star, inflammatory infiltrate; *n* = 3. **D** The ultrastructure of the intestine of juvenile yellow catfish was observed by transmission electron microscopy. N, nucleus; M, mitochondria; ER, endoplasmic reticulum; Tj, tight junctions; Mv, microvilli; Muc, mucus cells; De, desmosomes; Red arrow: endoplasmic reticulum expansion; Red star: obvious vacuolation; Green star: internal vacuolation, membrane damage, and myeloid lesions

As shown in Fig. 1B, in contrast to the PBS group, the DSS + NP group exhibited a lower survival rate and increased enteritis mortality (*P* < 0.05). Compared to the DSS + NP group, the DSS + ECP0 group demonstrated a significantly higher incidence of enterocolitis (*P* < 0.05). As shown in Fig. 1C and D, the intestinal mesenteric epithelium of DSS + NP group was vacuolized, with blurred tight junctions, localized cytoplasmic vacuolization, vacuolization of the inner organelles, membrane damage, and medullary lesions compared to the PBS group. The DSS + ECP0 group exhibited mitochondrial vacuolization, severe expansion of the endoplasmic reticulum, and sparse and disorganized microvilli when compared to the DSS + NP group.

#### Immunoreactive substances

As shown in Table 1, in contrast to the PBS group, the DSS + NP group exhibited a substantial reduction in LZM and ACP activity (*P* < 0.05) and a significant elevation in MPO activity of the intestine (*P* < 0.05). Comparing the DSS + ECP0 group to the DSS + NP group, the enzymatic activities of LZM, C3, and C4 were considerably reduced (*P* < 0.05).

**Table 1:** Immune activity substances in the intestine of juvenile yellow catfish after DSS-induced enteritis

Item	PBS	Dietary enzymatic cottonseed protein levels, %							*P*-value		
		42% CP	39% CP								
		0 (NP)	0 (ECP0)	1 (ECP1)	2 (ECP2)	3 (ECP3)	4 (ECP4)	5 (ECP5)	ANOVA	Linear	Quadratic
MPO	1.70 ± 0.16^t^	3.30 ± 0.28	3.19 ± 0.31^d;ns^	2.18 ± 0.18^b^	1.72 ± 0.15^a;#^	2.64 ± 0.28^c^	4.32 ± 0.41^e^	5.60 ± 0.50^f^	< 0.001	< 0.001	< 0.001
LZM	81.31 ± 8.48^t^	126.23 ± 11.58	103.89 ± 9.86^b;*^	142.94 ± 14.27^c^	213.40 ± 20.52^d;#^	102.41 ± 5.02^ab^	88.44 ± 8.90^a^	99.54 ± 5.60^ab^	< 0.001	< 0.001	< 0.001
C3	33.42 ± 2.07^ns^	34.16 ± 0.56	24.42 ± 1.37^a;*^	26.35 ± 2.06^b^	31.18 ± 1.28^c;#^	33.07 ± 1.46^d^	31.59 ± 1.04^ cd^	30.82 ± 0.96^c^	< 0.001	< 0.001	< 0.001
C4	2.55 ± 0.30^ns^	2.49 ± 0.28	1.88 ± 0.18^a;*^	2.69 ± 0.31^b^	2.95 ± 0.27^b;#^	4.76 ± 0.45^c^	2.87 ± 0.27^b^	2.22 ± 0.16^a^	< 0.001	< 0.001	< 0.001
ACP	148.51 ± 14.57^t^	121.39 ± 11.94	126.23 ± 8.60^ab; ns^	135.50 ± 14.08^bc^	143.40 ± 10.65^c;ns^	136.24 ± 12.39^bc^	135.41 ± 9.87^bc^	117.37 ± 12.36^a^	0.009	0.197	< 0.001

*MPO* Neutrophils express myeloperoxidase, U/g wet weight, *LZM* Lysozyme, U/mg prot, *C3* Complement component 3, mg/g prot, *C4* Complement component 4, mg/g prot, *ACP* Acid phosphatase, King unit/g prot

Date are presented as the mean ± standard deviation (*n* = 6). One-way ANOVA was used for data from the DSS + ECP0–5 groups, and mean values within the same row with different superscripts were significantly different (*P* < 0.05). Data from the DSS + NP group were compared with those from the PBS, DSS + ECP0, and DSS + ECP2 groups using independent samples *t*-test. ^t^Indicates a significant difference between the PBS group and DSS + NP group (*P* < 0.05); *Indicates a significant difference between the DSS + NP group and ECP0 group (*P* < 0.05); ^#^Indicates a significant difference between the DSS +NP group and DSS + ECP2 group (*P* < 0.05); ^ns^Indicates no significant between DSS + NP group and DSS + ECP0 group and DSS + ECP2 group (*P* > 0.05)

#### Macrophage polarization and its associated pathways

**Fig. 2:**
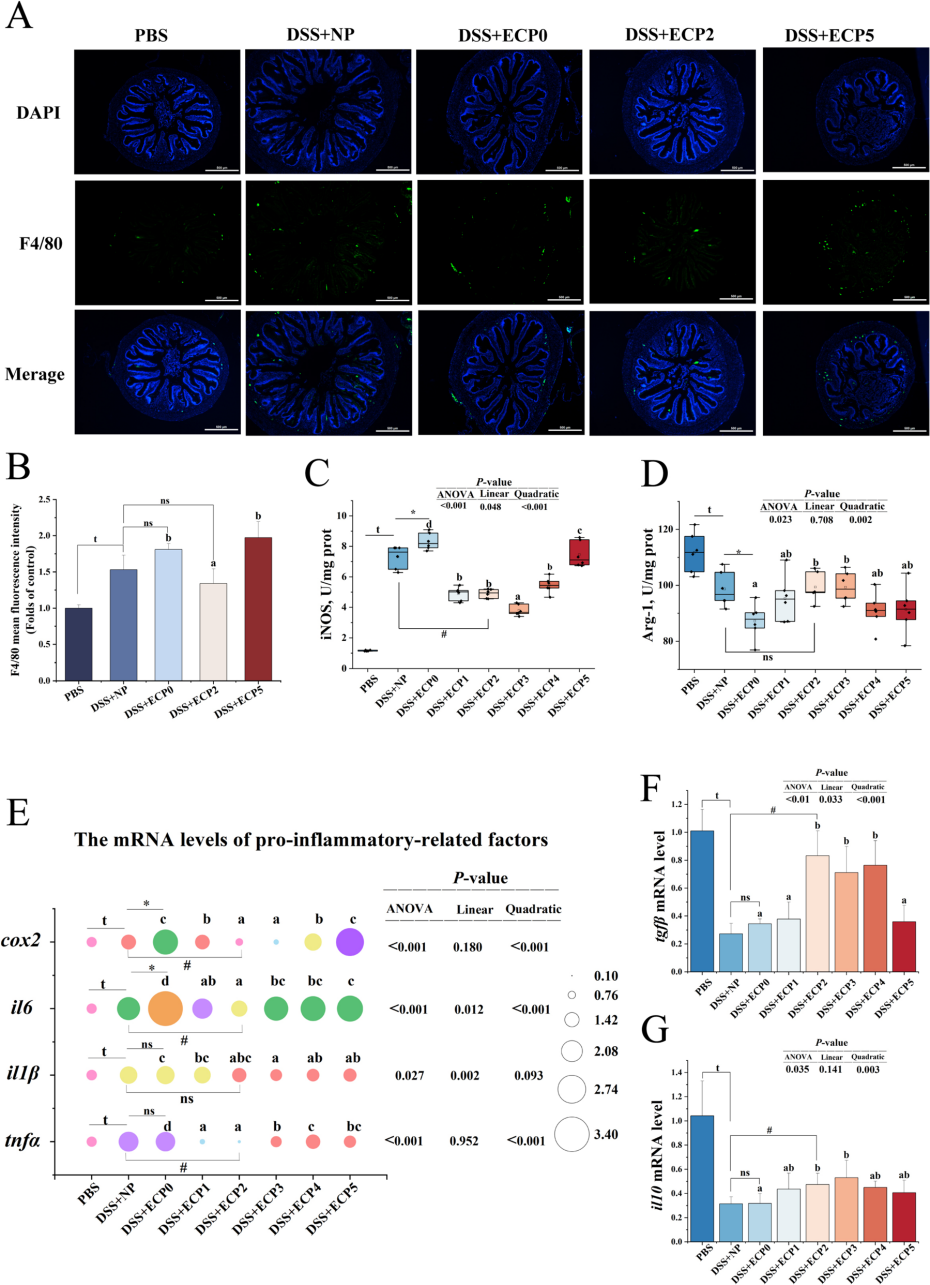
Intestinal macrophages and the polarization of macrophages in juvenile yellow catfish after DSS-induced enteritis. **A** and **B** Immunofluorescence and statistical results of F4/80, magnification 100 ×, scale bar = 500 μm (*n* = 3). **C** Inducible nitric oxide synthase (iNOS), M1 macrophage surface marker, *n* = 6. **D** Arginase 1 (Arg-1), M2 macrophage surface marker (*n* = 6). **E** The mRNA levels of the pro-inflammatory cytokine (*n* = 6). **F** The mRNA levels of transforming growth factor β (*tgfβ*) (*n* = 6) **G** The mRNA levels of interleukin-10 (*il10*) (*n* = 6). One-way ANOVA was used for data from the DSS + ECP0–5 groups, and mean values with different superscripts were significantly different (*P* < 0.05). Data from the DSS + NP group were compared with those from the PBS, DSS + ECP0, and DSS + ECP2 groups using independent samples *t*-test, ^t^Indicates a significant difference between the PBS group and DSS + NP group (*P* < 0.05); *Indicates a significant difference between the DSS + NP group and ECP0 group (*P* < 0.05); ^#^ Indicates a significant difference between the DSS + NP group and DSS + ECP2 group (*P* < 0.05); ^ns^Indicates no significant between DSS + NP group and DSS + ECP0 group and DSS + ECP2 group (*P* > 0.05)

As shown in Fig. 2, the mean fluorescence intensity of F4/80 (*P* < 0.05), the enzymatic activities of iNOS, the mRNA levels of *il1β*, cyclooxygenase-2 (*cox2*), *il6*, *tnfα* were considerably increased (*P* < 0.05); the enzymatic activities of Arg-1, the mRNA levels of *il10* and *tgfβ* were significantly decreased (*P* < 0.05) in the DSS + NP group, compared to the PBS group. In the DSS + ECP0 group, the enzymatic activities of iNOS and the mRNA levels of *cox2* and *il6* were remarkably elevated (*P* < 0.05), the enzymatic activity of Arg-1 was significantly reduced (*P* < 0.05), in contrast to the DSS + NP group.

**Fig. 3:**
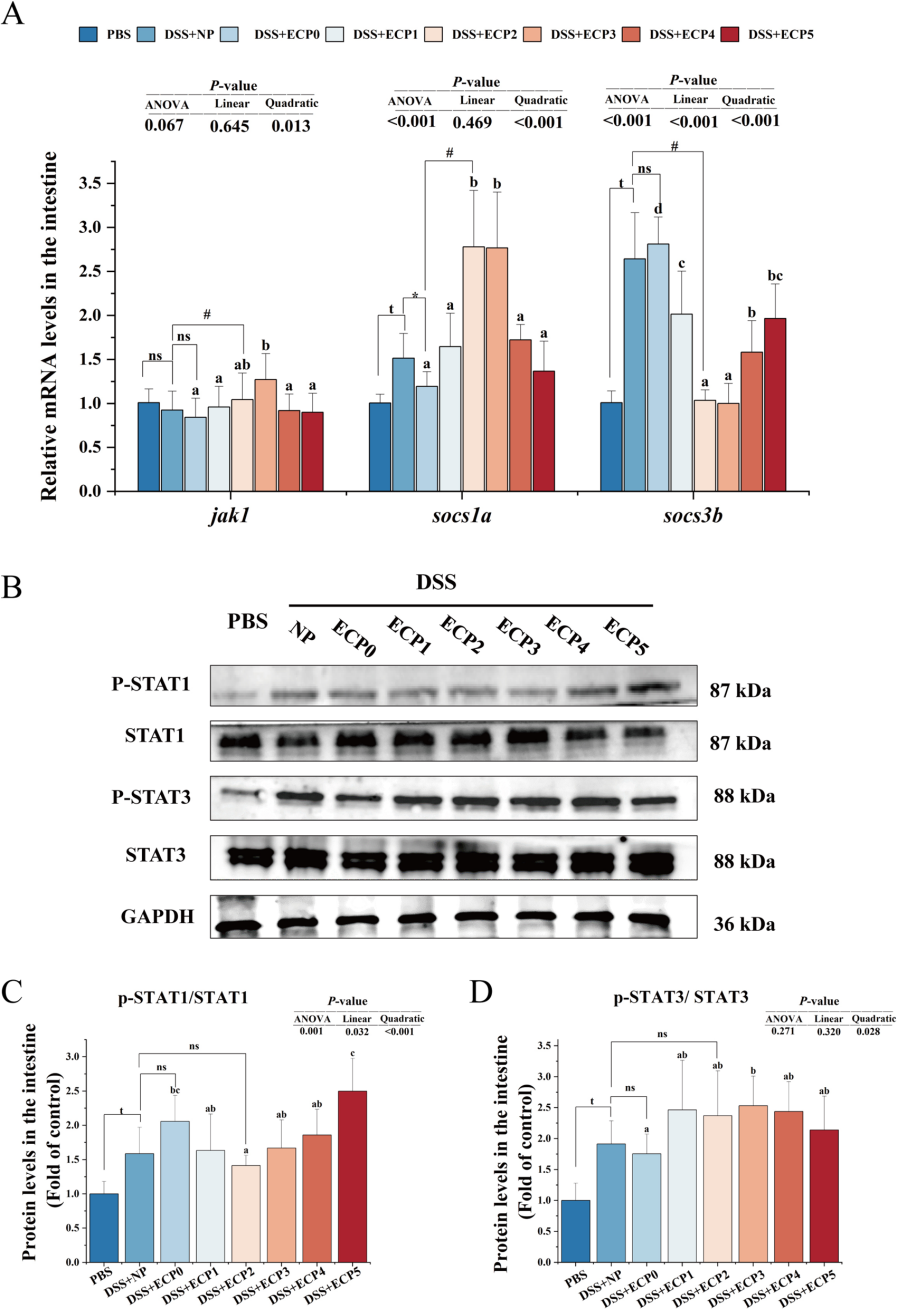
JAK/STAT signaling pathway in the intestine of juvenile yellow catfish after DSS-induced enteritis. **A** Expression levels of JAK/STAT pathway-associated genes. **B**–**D** The protein expression of p-STAT1 and p-STAT3 (*n* = 6). p-STAT1, phosphorylated-signal transducer and activator of transcription 1; STAT1, signal transducer and activator of transcription 1; P-STAT3, phosphorylated-signal transducer and activator of transcription 3; STAT3, signal transducer and activator of transcription 3. One-way ANOVA was used for data from the DSS + ECP0–5 groups, and mean values with different superscripts were significantly different (*P* < 0.05). Data from the DSS + NP group were compared with those from the PBS, DSS + ECP0, and DSS + ECP2 groups using independent samples *t*-test. ^t^Indicates a significant difference between the PBS group and DSS + NP group (*P* < 0.05); ^*^Indicates a significant difference between the DSS + NP group and ECP0 group (*P* < 0.05); ^#^Indicates a significant difference between the DSS + NP group and DSS + ECP2 group (*P* < 0.05); ^ns^Indicates no significant between DSS + NP group and DSS + ECP0 group and DSS + ECP2 group (*P* > 0.05)

In the DSS + NP group, the mRNA levels of suppressor of cytokine signaling 1a (*socs1a*) and suppressor of cytokine signaling 3b (*socs3b*) (Fig. 3 A), the protein expression levels of phosphorylated signal transducer and activator of transcription 1 (p-STAT1) relative to total STAT1 and p-STAT3 relative to total STAT3 (Fig. 3B–D) were considerably increased (*P* < 0.05), when compared in the PBS group.

#### Necroptosis and its associated pathways

**Fig. 4:**
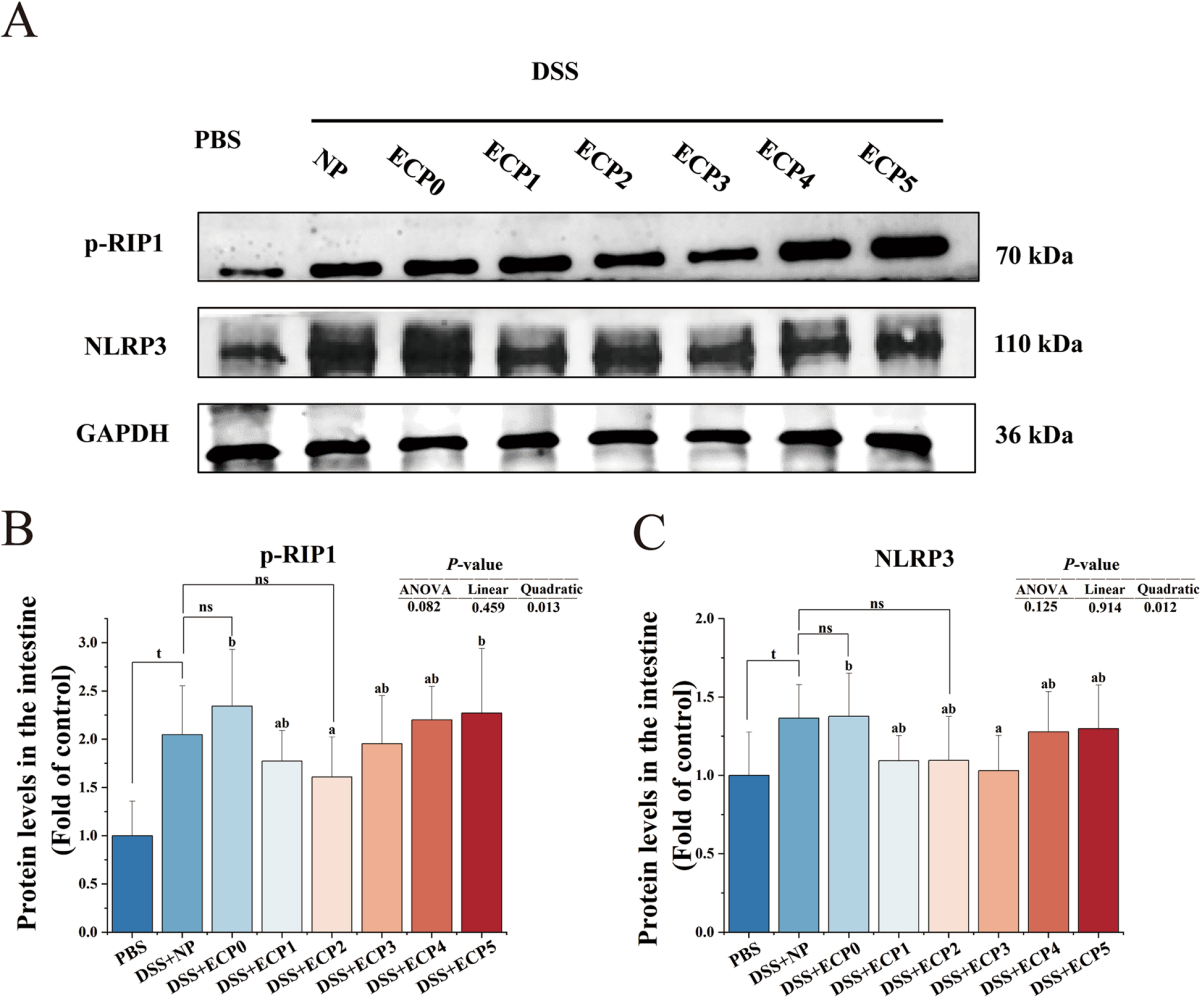
The levels of p-RIP1 and NLRP3 protein in the intestine of juvenile yellow catfish after DSS-induced enteritis (*n* = 6). p-RIP1, phosphorylated receptor-interacting protein 1; NLRP3, NLR-family pyrin domain-containing protein 3. One-way ANOVA was used for data from the DSS + ECP0–5 groups, and mean values with different superscripts were significantly different (*P* < 0.05). Data from the DSS + NP group were compared with those from the PBS, DSS + ECP0, and DSS + ECP2 groups using independent samples *t*-test, ^t^Indicates a significant difference between the PBS group and DSS + NP group (*P* < 0.05); ^ns^Indicates no significant between DSS + NP group and DSS + ECP0 group and DSS + ECP2 group (*P* > 0.05)

**Fig. 5:**
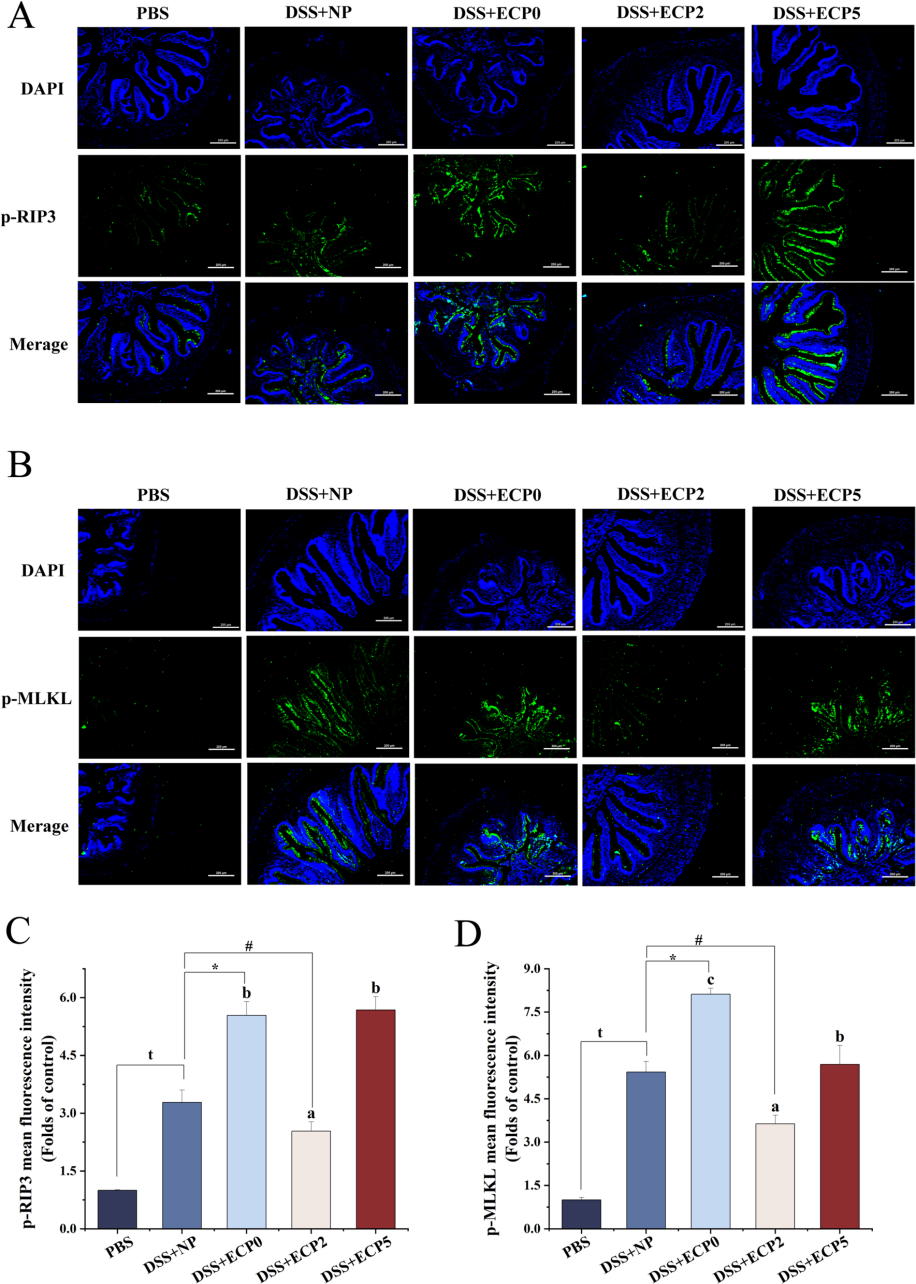
The levels of p-RIP3 and p-MLKL in the intestine of juvenile yellow catfish after DSS-induced enteritis. **A** and **C** Immunofluorescence and statistical results of phosphorylated receptor-interacting protein 3 (p-RIP3). **B** and **D** Immunofluorescence and statistical results of phosphorylated mixed lineage kinase domain-like (p-MLKL). Magnification 200 ×, scale bar = 200 μm, *n* = 3. One-way ANOVA was used for data from the DSS + ECP0, DSS + ECP2, and DSS + ECP05 groups, and mean values with different superscripts were significantly different (*P* < 0.05). Data from the DSS + NP group were compared with those from the PBS, DSS + ECP0, and DSS + ECP2 groups using independent samples *t*-test. ^t^Indicates a significant difference between the PBS group and DSS + NP group (*P* < 0.05); ^*^Indicates a significant difference between the DSS + NP group and ECP0 group (*P* < 0.05); ^#^Indicates a significant difference between the DSS + NP group and DSS + ECP2 group (*P* < 0.05)

The DSS + NP group exhibited a marked upregulation (*P* < 0.05) in the protein expression of phosphorylated-RIP1 (p-RIP1) and NLR-family pyrin domain-containing protein 3 (NLRP3) (Fig. 4), the mean fluorescence intensity of phosphorylated-RIP3 (p-RIP3) and phosphorylated-MLKL (p-MLKL) (Fig. 5), compared to the PBS group. The mean fluorescence intensity of p-RIP3 and p-MLKL (Fig. 5) was significantly higher (*P* < 0.05) in the DSS + ECP0 group than in the DSS + NP group.

**Fig. 6:**
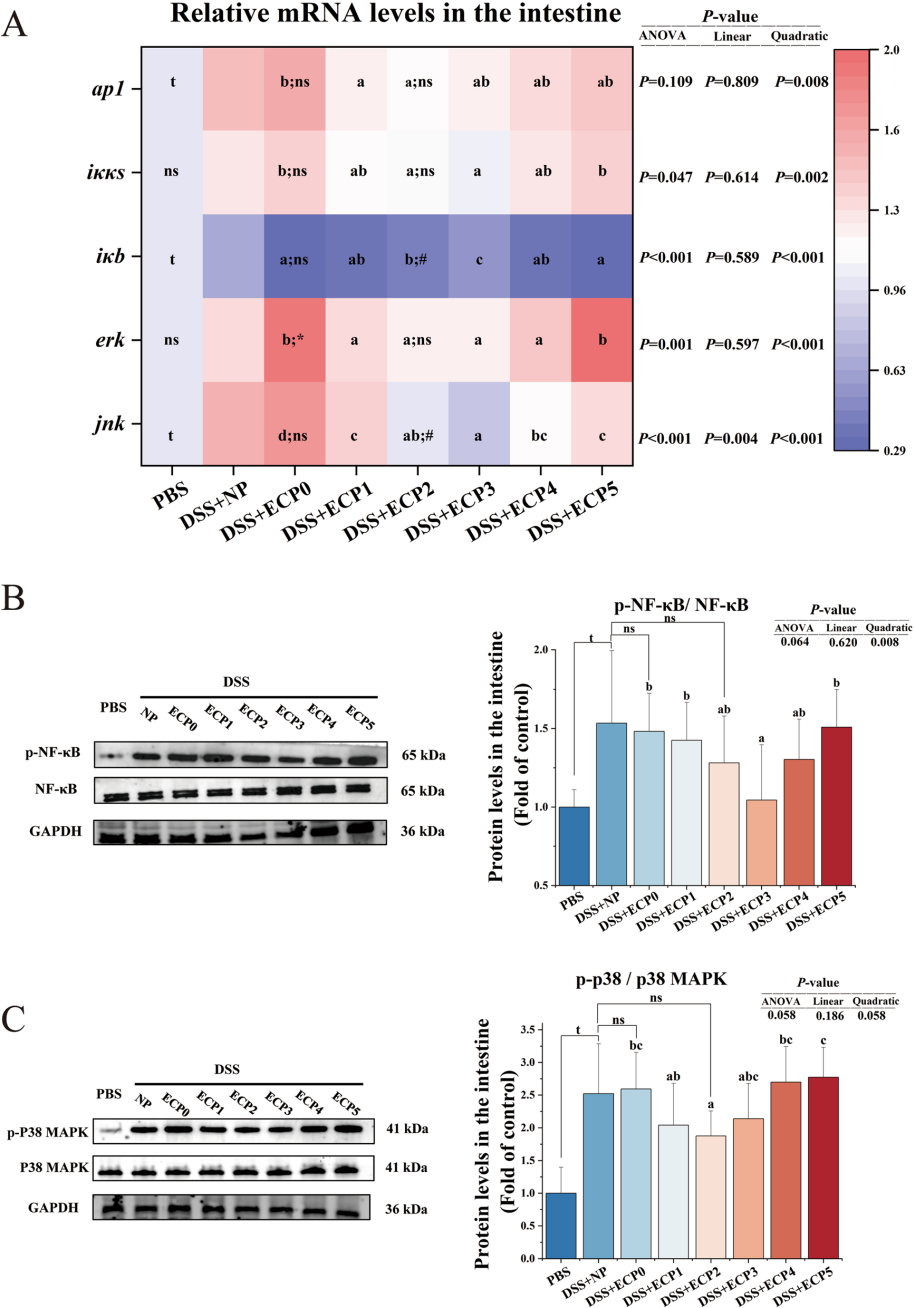
ECP activated NF-κB and MAPK signaling pathways in juvenile yellow catfish intestine after DSS-induced enteritis. **A** The mRNA levels of the relative of NF-κB and MAPK signaling pathway. **B** The protein expression of phosphorylated-nuclear factor kappa-B (p-NF-κB). **C** The protein expression of phosphorylated-P38 mitogen-activated protein kinase (p-p38 MAPK) (*n* = 6). One-way ANOVA was used for data from the DSS + ECP0–5 groups, and mean values with different superscripts were significantly different (*P* < 0.05). Data from the DSS + NP group were compared with those from the PBS, DSS + ECP0, and DSS + ECP2 groups using independent samples *t*-test. ^t^Indicates a significant difference between the PBS group and DSS + NP group (*P* < 0.05); ^*^Indicates a significant difference between the DSS + NP group and ECP0 group (*P* < 0.05); ^#^Indicates a significant difference between the DSS + NP group and DSS + ECP2 group (*P* < 0.05); ^ns^Indicates no significant between DSS + NP group and DSS + ECP0 group and DSS + ECP2 group (*P* > 0.05)

In the DSS + NP group, the mRNA levels of c-Jun N-terminal kinase (*jnk*), and activator protein 1 (*ap1*) (Fig. 6A) were considerably increased (*P* < 0.05), and the protein expression levels of phosphorylated p38 (p-p38) relative to total p38 mitogen-activated protein kinase (MAPK) and phosphorylated NF-κB (p-NF-κB) relative to total NF-κB were also enhanced (*P* < 0.05) (Fig. 6B), the mRNA of inhibitor of NF-κB (*iκb*) (Fig. 6A) was significantly decreased (*P* < 0.05), compared to the PBS group. The mRNA level of extracellular-signal-regulated kinase (*erk*) (Fig. 6A) was significantly higher (*P* < 0.05) in the DSS + ECP0 group than in the DSS + NP group.

### Effects of ECP addition to LP diets on DSS-induced enteritis in juvenile yellow catfish

#### Enteritis morbidity, mortality rate, and intestinal organization

The DSS + ECP1–5 groups exhibited significantly lower enteritis morbidity (*P* < 0.05) compared to the DSS + ECP0 group, while the DSS + ECP2 group had the lowest morbidity. The survival rate demonstrated a dose-dependent increase with the greater significant proportion of dietary ECP supplementation, reaching 100% in the DSS + ECP2–5 groups (Fig. 1B). The DSS + ECP2 and DSS + ECP5 groups displayed fewer intestinal blebs, reduced sites of inflammatory infiltration, decreased epithelial vacuolization, relatively well-defined tight junctions, a more intact microvilli arrangement, and reduced intracellular vacuolization of organelles compared to the DSS + ECP0 group (Fig. 1 C and D).

#### Immunoreactive substances

As illustrated in Table 1, there were several significant changes in enzyme activity and content among different groups. In the DSS + ECP1–3 groups, MPO enzyme activity was notably lower (*P* < 0.05) than in the DSS + ECP0 group. The LZM enzyme activity was substantially higher (*P* < 0.05) in the DSS + ECP1–2 groups, while the ACP enzyme activity was significantly higher (*P* < 0.05) in the DSS + ECP2 group, compared with the DSS + ECP0 group. The C3 content in the DSS + ECP2–5 groups and the C4 content in the DSS + ECP1–4 groups showed significant increases (*P* < 0.05) compared to the DSS + ECP0 group.

#### Macrophage polarization and its associated pathways

As depicted in Fig. 2, the iNOS enzyme activity, the mRNA levels of *il6* and *tnfα* in the DSS + ECP1–5 groups, the mRNA level of *cox2* in the DSS + ECP1–4 groups, the mRNA level of *il1β* in the DSS + ECP3–5 groups, and the protein expression level of p-STAT1/STAT1 and the mean fluorescence intensity of F4/80 in the DSS + ECP2 group were all remarkably decreased (*P* < 0.05) contrasted to the DSS + ECP0 group. The Arg-1 enzyme activity in the DSS + ECP3 group, the mRNA levels of *tgfβ* in the DSS + ECP2–4 groups, and the mRNA levels of *il10* in the DSS + ECP2–3 groups were significantly increased (*P* < 0.05) compared to the DSS + ECP0 group. The mRNA levels of *socs1a* in the DSS + ECP2–3 groups, and the mRNA levels of Janus kinase 1 (*jak1*) and p-STAT3/STAT3 protein expression levels in the DSS + ECP3 group were significantly increased (*P* < 0.05) compared to the DSS + ECP0 group, as shown in the Fig. 3.

#### Necroptosis and its associated pathways

As shown in Figs. 4 and 5, the intestinal p-RIP1 protein expression levels, the average fluorescence intensity of p-RIP3 and p-MLKL in the DSS + ECP2 group, and the protein expression levels of NLRP3 in the DSS + ECP3 group were all remarkably decreased (*P* < 0.05) compared to those of the DSS + ECP0 group. In Fig. 6, the mRNA levels of *erk* in the DSS + ECP1–4 groups, the mRNA levels of *jnk* in the DSS + ECP1–5 groups, the mRNA levels of *ap1* and the p-p38/p38 MAPK protein expression levels in the DSS + ECP2 group, the mRNA levels of inhibitor of kappa B kinase (*iκκs*) in the DSS + ECP2–3 groups, the protein expression levels of p-NF-κB/NF-κB in the DSS + ECP3 group, were all remarkably decreased (*P* < 0.05) than those of the DSS + ECP0 group. The mRNA level of *iκb* was significantly increased (*P* < 0.05) in the DSS + ECP2–3 groups compared with the DSS + ECP0 group.

### Effect of adding 2% ECP to LP diets on DSS-induced enteritis in juvenile yellow catfish compared to the NP diets

The DSS + ECP2 group demonstrated a higher survival rate and lower enteritis morbidity than the DSS + NP group (Fig. 1B). MPO activity and C3 content (Table 1) were significantly reduced (*P* < 0.05), although C4 content, LZM, and ACP activity (Table 1) were significantly higher (*P* < 0.05) in the DSS + ECP2 group compared to the DSS + NP group. The DSS + ECP2 group exhibited markedly higher mRNA levels of *tnfα*, *il10*, *jak1*, and *socs1a*, along with decreased enzymatic activity of iNOS and mRNA levels of *cox2*, *il6*, *tnfα*, and *socs3b* compared to the DSS + NP group, as shown in Figs. 2 and 3. The mean fluorescence intensity of p-RIP3 and p-MLKL was significantly lower (*P* < 0.05) in the DSS + ECP2 group than in the DSS + NP group (Fig. 5). Additionally, the mRNA levels of *iκb* and *jnk* were notably reduced in the DSS + ECP2 group compared to the DSS + NP group (Fig. 6).

## Discussion

### DSS-induced enteritis lead to an intestinal inflammatory response

DSS is a well-established chemical irritant commonly utilized to model intestinal inflammation. Previous studies have demonstrated that DSS-induced detrimental effects on intestinal epithelial cells and mucosal barrier integrity [23, 29]. This study showed that enteritis caused by DSS in juvenile yellow catfish changes the polarization of macrophages by promoting the polarization of M1 macrophages and inhibiting that of M2 macrophages. Additionally, necroptosis is induced, leading to damage in intestinal epithelial cells.

### Enteritis was exacerbated by a 3% reduction in dietary protein levels

Protein is a crucial component of aquatic animal feed, significantly influencing the growth, development, and physiological health of fish [30]. Our previous research demonstrated that a reduction in dietary protein content by 3% resulted in a decline in growth performance, specifically in specific growth rate (SGR) and feed intake (FI), among juvenile yellow catfish [8]. In the current study, we observed that a 3% reduction in dietary protein levels also led to an increase in enteritis-related mortality and exacerbated intestinal damage following DSS-induced enteritis. These collective observations suggest dietary protein levels play a dual regulatory role in maintaining both growth performance and intestinal barrier function. Moreover, a 3% reduction in dietary protein levels resulted in a decrease in the content (C3 and C4) and enzyme activity (LZM) of immunoreactive substances. M1-type macrophage surface markers (iNOS enzyme activity, the mRNA of *il6* and *cox2*) and the mRNA levels of the macrophage polarization-associated molecule *socs1a* exhibited an increase, and the mRNA levels of the M2-type macrophage surface marker *il10* exhibited a decrease. Additionally, our findings revealed enhanced activation of necroptosis-related signaling pathways, characterized by an upregulation of necroptosis-associated signaling molecules (p-RIP1 and p-MLKL) and an increase in mRNA expression of the necroptosis-associated molecule *erk*. These results demonstrated that a 3% reduction in dietary protein exacerbates DSS-induced enteritis in juvenile yellow catfish. Studies have shown that lower dietary protein levels may cause intestinal inflammation in juvenile blotched snakeheads (*Channa maculata*) [31] and compromise mucosal humoral immunity in the colon of pigs [32], which was consistent with our findings.

Ultimately, reduced dietary protein may worsen damage to the intestinal immune system in animals, leading to more significant harm to epithelial cells, increased polarization of inflammatory macrophages (M1), decreased polarization of anti-inflammatory macrophages (M2), and heightened necroptosis.

### The addition of ECP to LP diets alleviates enteritis

#### ECP reduced enteritis morbidity and mortality and mitigates damage to intestinal tissue structure

An animal's resistance to illness may display visually as enteritis morbidity and survival rate [33]. ECP supplementation decreased enteritis morbidity and increased the survival rate of juvenile yellow catfish after DSS-induced enteritis in our study, indicating that dietary ECP may enhance intestinal immunity in fish. The structural integrity of the intestinal mucosa is an essential measure of the inflammatory damage [34]. According to our findings, adding dietary ECP decreased the development of inflammatory infiltrates, structural vacuolization, tight junction blurring, sparse microvilli, internal vacuolization of organelles, membrane damage, and myeloid lesions caused by DSS. These histology findings indicated that ECP reduced inflammation and repairs intestinal barrier damage brought on by DSS-induced enteritis. This aligns with previous results where 2% ECP in LP diets improved growth and intestinal structure in juvenile yellow catfish [8]. These consistent protective effects suggest that ECP may influence key pathways related to intestinal health.

#### ECP promoted the production of immunoreactive substances

MPO is a peroxidase that mediates the inflammatory response in several disorders and is most frequently expressed in neutrophils [35]. Our research indicated that adding 1%−3% ECP to LP diets decreased MPO activity, suggesting that ECP exerts anti-inflammatory effects and alleviates enteritis. This soothing effect could be related to an increase in immunoreactive substances. Antimicrobial peptides, lysozyme, and complement are present in fish mucosal secretions, which are essential components of the immune system and are involved in phagocytosis, antigen destruction, inflammatory response, and tissue damage healing [36]. LZM, C3, C4, and ACP activities were increased in this study when 2% ECP was included in LP diets. These findings align with previous research showing that cotton meal protein hydrolysate enhanced the activity of ACP, AKP, and LZM in the hemolymph of the Chinese mitten crab (*Eriocheir sinensis*) [37]. In conclusion, dietary supplementation with ECP can enhance the synthesis of immunoreactive substances in aquatic animals, potentially improving their immune competence.

#### ECP modulated macrophage polarization

The inflammatory response becomes excessively activated, leading to sustained tissue damage primarily attributed to aberrant signaling pathways and dysfunctional expression in macrophages [38]. Mature macrophages can be labeled by F4/80 [39]. This investigation found that adding 2% ECP to the diet decreased the mean fluorescence intensity of F4/80, indicating that dietary ECP could reduce macrophage infiltration. The two polarizations of mature macrophages are "classically activated" or pro-inflammatory (M1) and "selectively activated" or anti-inflammatory (M2) [40]. While M2 macrophages are characterized by the promotion of Arg-1 synthesis and the secretion of anti-inflammatory cytokines, such as IL-10 and TGF-β, M1 macrophages are defined by the production of iNOS, COX2, and high levels of pro-inflammatory cytokine release, including TNF-α, IL-6, and IL-1β [41, 42]. Moreover, polarized macrophages in teleost fish exhibit an inflammatory phenotype similar to that in mammals [43]. In this research, adding 2%−3% ECP to the diet dramatically reduced iNOS activity and mRNA levels for *cox2*, *il6*, *il1β*, and *tnfα* while significantly increasing Arg-1 activity and mRNA levels for *il10* and *tgfβ*. Based on these results, dietary ECP stimulated M2-type macrophage polarization while suppressing M1-type macrophage polarization.

JAK signaling and the activator of transcription (STAT) signaling pathways have been intensively studied and shown to regulate the production of a wide range of inflammatory molecules, which play crucial roles in intestinal inflammation [44, 45]. Studies have shown that JAK-STAT signaling regulates the phenotype and activity of macrophages [46, 47]. In addition, the JAK-STAT pathway is used by the SOCS family of proteins to block the negative feedback of cytokine-induced signaling. Inhibition of SOCS1 leads to STAT1 signaling, which polarizes M1-type macrophages, and inhibition of SOCS3 leads to STAT3 signaling, which polarizes M2-type macrophages [48, 49]. In our investigation, 3% ECP supplementation markedly raised the mRNA levels of *jak1*, *socs1a*, and the p-STAT3/STAT3 protein expression, while 2% ECP markedly reduced the mRNA levels of *socs3b* and p-STAT1/STAT1 protein expression. These results suggested that ECP might modulate macrophage polarization by potentially activating the SOCS/JAK/STAT signaling pathway, thereby inhibiting M1 phenotype while promoting M2 polarization. However, the precise regulatory relationship between ECP and the SOCS/JAK/STAT pathway requires further experimental validation through genetic approaches such as knockout studies or pharmacological interventions.

#### ECP ameliorated necroptosis

Necroptosis is recognized as a pro-inflammatory reaction associated with the onset of inflammation [50]. Numerous investigations have demonstrated a strong correlation between necroptosis in intestinal epithelial cells and colitis induced by DSS [26, 51]. Necroptosis is initiated by immune ligands, and RIPK1 and RIPK3 bind through the receptor homology domain (RHD) to create necrotic vesicles; this activation of RIPK3 subsequently phosphorylates MLKL to activate it further [52]. In the end, phosphorylated MLKL disrupts cellular integrity by translocating to the inner leaflet of the plasma membrane [53]. In this investigation, 2% ECP supplementation dramatically lowered p-RIP1 protein expression and p-RIP1 and p-MLKL average fluorescence intensity, suggesting that ECP can alleviate DSS-induced necroptosis. Furthermore, it has been demonstrated that RIP3 activation triggers the NLRP3 inflammasome pathway [54]. NLRP3 is a cytoplasmic multiprotein complex whose overactivation has been associated with the etiology of several inflammatory illnesses [55]. In our investigation, 3% ECP supplementation significantly decreased NLRP3 protein expression. According to the results above, ECP effectively decreased necroptosis by inhibiting the RIP/MKLK-NLRP3 pathway.

The transcription factor NF-κB is a vital mediator in the inflammatory response and regulates various aspects of innate and adaptive immune function [56]. Activation of the Iκκ complex induces the phosphorylation of IκB-α, which deactivates the nuclear transcription factor NF-κB, responsible for regulating the production of pro-inflammatory cytokines [57]. Numerous studies have demonstrated that suppressing the NF-κB pathway effectively reduces necroptosis [58, 59]. According to our research, adding 3% ECP to the diet significantly increased the mRNA levels of *iκb* while dramatically lowering the levels of p-NF-κB/NF-κB protein and the mRNA of *iκks*. Furthermore, enzymatic cottonseed protein concentrate decreased the mRNA levels of *nf-κb* in the liver of largemouth bass (*Micropterus salmoides*) [17], aligning with our findings. These findings suggested that ECP may attenuate inflammation and necroptosis by suppressing the NF-κB signaling pathway. However, whether ECP directly modulates NF-κB activation remains to be experimentally validated, necessitating further mechanistic investigations.

Several investigations have shown that inhibiting the MAPK pathway can reduce necroptosis [60, 61]. Specifically, it has been demonstrated that RIPK1 and RIPK3 expression in RGE cells is significantly reduced by p38 inhibitors [62]. Furthermore, applying an extracellular signal-regulated kinase (ERK) inhibitor effectively decreases p-MLKL expression [63]. In a rat model of ischemia/reperfusion (I/R)-induced brain injury, the suppression of JNK has been shown to attenuate necroptosis by downregulating RIPK3 expression [64]. In the present study, dietary supplementation with 2% ECP reduced the mRNA levels of *erk* and *jnk* and the protein expression of p-p38/p38MAPK, suggesting that ECP modulated the AMPK pathway. Additionally, AP-1 has been identified as a key mediator of zVAD-induced necroptosis in L929 cells, acting through the PKC-MAPKs-AP-1 signaling cascade [65]. Our findings reveal that dietary supplementation with 2% ECP significantly decreased the mRNA level of *ap1*. These findings suggested that ECP may exert its anti-necroptotic effects by inhibiting the p38 MAPK-AP-1 signaling pathway. However, our current experimental evidence demonstrated only an association rather than a causal relationship between ECP and p38 MAPK-AP-1 signaling. Further mechanistic studies will be required to establish this regulatory axis definitively.

### Compared to 2% ECP, treatment with 5% ECP reduced immunoreactive substances, promoted M2 macrophage polarization, and enhanced necroptosis

In the present study, the addition of 5% ECP to LP diets reduced immunoreactive substances (LZM, C4, and ACP), promoted M2 macrophage polarization (iNOS, *cox2*, *il6*, and *tnfα*), and enhanced necroptosis (p-RIP1, p-RIP3, and p-MLKL). The reasons are analyzed: after enzymatic treatment, cottonseed protein contains a small amount of gossypol [66]. Compared to the group with 10% cottonseed meal and rapeseed meal mixed vegetable protein (free gossypol: 24.77 mg/kg), using more than 10% of this mixture to replace fishmeal inhibited the growth and improved the expression of the inflammation factor (*il1β*, *il6*, *il8*, and *tnfα*) in liver of juvenile yellow catfish [67]. Therefore, we hypothesized that the adverse effects of adding 5% ECP (free gossypol: 35 mg/kg) on reduced immunoreactive substances, promoted M2 macrophage polarization, and enhanced necroptosis of juvenile yellow catfish may be due to higher levels of gossypol in the diet. However, this hypothesis requires further investigation.

### DSS-induced enteritis was relieved by adding 2% ECP after a 3% decrease in dietary protein levels, bringing them back to or even better than normal protein levels

The following alterations occurred during this investigation when 2% ECP was added after a 3% decrease in dietary protein levels: reduced MPO activity and elevated levels of immunoreactive substances (LZM, C4, and ACP) activity or levels; decreased activity and mRNA levels of surface markers (iNOS) and related cytokines (*cox2*, *il6*, and *tnfα*) in M1 macrophages, increased mRNA levels of associated cytokines (*il10* and *tgfβ*) in M2 macrophages, and elevated mRNA levels of pathways controlling macrophage polarization, including *socs1a* and *jak1*; in the necroptosis pathway, the mean fluorescence intensity of p-RIP3 and p-MLKL decreased, while the corresponding passages showed an increase in *iκb* mRNA levels and a decrease in *jnk* mRNA levels. These findings implied that, by altering macrophage polarization and necroptosis, 2% ECP added after a 3% decrease in dietary protein levels can return DSS-induced enteritis to or above normal protein levels. The observed effect could be explained by the fact that ECP primarily consists of small bioactive peptides, which are more efficiently digested, absorbed, and metabolized by fish. Our earlier findings demonstrated a 5% improvement in crude protein apparent digestibility in the ECP2 group compared to the NP group [8], which supports this opinion. Collectively, these findings suggested that 2% ECP supplementation can conserve dietary protein while meeting the requirements for intestinal immunity and development.

## Conclusions

This study demonstrates that DSS-induced enteritis in juvenile yellow catfish worsened when dietary protein is reduced by 3%. Adding ECP to LP diets significantly alleviates DSS-induced enteritis in juvenile yellow catfish through a dual mechanism: regulation of macrophage polarization and inhibition of necroptosis. On one hand, ECP supplementation reduces M1 macrophage polarization while promoting M2 macrophage polarization, potentially via the JAK/STAT signaling pathway. On the other hand, it suppresses necroptosis by inhibiting the phosphorylation of RIP1, RIP3, and MLKL, which may occur through the NF-κB and MAPK signaling pathways. Notably, adding 2% ECP to diets with a 3% protein reduction restores intestinal immune function to levels seen with normal protein and even provided greater protective effects. This study clarifies the protein-sparing efficacy of ECP and its immunomodulatory properties, thereby providing a strong theoretical foundation for the potential use of ECP.

## Supplementary Information

Supplementary Material 1: Table S1. Composition and nutrient contents of the diet; Table S2. The different peptides of the enzymatic cottonseed protein (ECP) as a proportion of total protein; Table S3. The primer sequences and accession numbers of genes were selected for analysis by real-time PCR; Table S4. Target proteins, dilution factor, antibody cat. no. and antibody source of proteins selected for analysis by western blotting.

Supplementary Material 2: Original Western blot images for all protein bands analyzed in the study.

## Data Availability

The datasets used and/or analyzed during the current study are available from the corresponding author upon reasonable request.

## Abbreviations

AP-1: Activator protein 1
Arg-1: Arginase 1
C3: Complement 3
C4: Complement 4
DSS: Dextran sulfate sodium
ECP: Enzymatic cottonseed protein
I/R: Ischemia/reperfusion
IκB: Inhibitor of NF-κB
IL-1β: Interleukin-1beta
IL-10: Interleukin-10
IL-6: Interleukin-6
iNOS: Inducible nitric oxide synthase
JAK1: Janus kinase 1
JNK: C-Jun N-terminal kinase
LZM: Lysozyme
MLKL: Mixed lineage kinase domain-like
MAPK: Mitogen-activated protein kinase
NF-κB: Nuclear factor kappa-B
NLRP3: NLR-family pyrin domain-containing protein 3
RIP1: Receptor-interacting protein 1
RIP3: Receptor-interacting protein 3
SOCS1: Suppressor of cytokine signaling 1
SOCS3: Suppressor of cytokine signaling 3
STAT1: Signal transducer and activator of transcription 1
STAT3: Signal transducer and activator of transcription 3
TGF-β: Transforming growth factor-beta
TNF-α: Tumor necrosis factor-alpha
