# Supplementary material for: Enzymatic cottonseed protein alleviates DSS-induced enteritis in juvenile yellow catfish (Pelteobagrus fulvidraco): focus on macrophage polarization and necroptosis in the intestine
Source: J Anim Sci Biotechnol. 2025 Aug 26;16:119. doi: 10.1186/s40104-025-01248-z (PMC12379407; doi:10.1186/s40104-025-01248-z)
Supplement: Supplementary file 1 — Supplementary Material 1: Table S1. Composition and nutrient contents of the diet; Table S2. The different peptides of the enzymatic cottonseed protein (ECP) as a proportion of total protein; Table S3. The primer sequences and accession numbers of genes were selected for analysis by real-time PCR; Table S4. Target proteins, dilution factor, antibody cat. no. and antibody source of proteins selected for analysis by western blotting. [file 40104_2025_1248_MOESM1_ESM.docx]

**Table S1**

Composition and nutrient contents of the diet.

| Ingredients% | 42%CP | 39%CP | | | | | |
| --- | --- | --- | --- | --- | --- | --- | --- |
|  | NP | ECP0 | ECP1 | ECP2 | ECP3 | ECP4 | ECP5 |
| Fish meal | 25.00 | 23.10 | 22.10 | 21.11 | 20.12 | 19.13 | 18.14 |
| Chicken meal | 15.00 | 13.80 | 13.80 | 13.80 | 13.80 | 13.80 | 13.80 |
| Soybean meal | 16.68 | 15.10 | 15.10 | 15.10 | 15.10 | 15.10 | 15.10 |
| Corn protein meal | 8.50 | 7.67 | 7.67 | 7.67 | 7.67 | 7.67 | 7.67 |
| Wheat flour | 15.00 | 15.00 | 15.00 | 15.00 | 15.00 | 15.00 | 15.00 |
| Soybean oil | 4.03 | 4.35 | 4.44 | 4.52 | 4.60 | 4.68 | 4.76 |
| Phospholipid oil | 2.00 | 2.00 | 2.00 | 2.00 | 2.00 | 2.00 | 2.00 |
| Microcrystalline cellulose | 0.20 | 4.91 | 4.76 | 4.63 | 4.47 | 4.30 | 4.14 |
| Carboxymethyl cellulose | 7.00 | 7.00 | 7.00 | 7.00 | 7.00 | 7.00 | 7.00 |
| Ca (H_2_PO_4_)_2_ | 1.22 | 1.43 | 1.45 | 1.45 | 1.48 | 1.52 | 1.55 |
| Mineral premix ^1^ | 2.00 | 2.00 | 2.00 | 2.00 | 2.00 | 2.00 | 2.00 |
| Vitamin premix ^2^ | 1.00 | 1.00 | 1.00 | 1.00 | 1.00 | 1.00 | 1.00 |
| Choline chloride premix ^3^ | 1.00 | 1.00 | 1.00 | 1.00 | 1.00 | 1.00 | 1.00 |
| Butylated hydroxyanisole (99%) | 0.015 | 0.015 | 0.015 | 0.015 | 0.015 | 0.015 | 0.015 |
| L-Lysine（78.8%） | 1.35 | 1.59 | 1.62 | 1.65 | 1.68 | 1.71 | 1.74 |
| L-Met（99%） | 0.00 | 0.04 | 0.05 | 0.06 | 0.07 | 0.08 | 0.09 |
| ECP | 0.00 | 0.00 | 1.00 | 2.00 | 3.00 | 4.00 | 5.00 |
| Nutrient content (%) |  |  |  |  |  |  |  |
| Crude protein^4^ | 42.46 | 39.36 | 39.24 | 39.97 | 39.62 | 39.59 | 39.72 |
| Crude lipid^4^ | 10.41 | 10.69 | 10.73 | 10.54 | 10.39 | 10.28 | 10.34 |
| Gross energy KJ/g^4^ | 18.48 | 18.26 | 18.22 | 18.30 | 18.29 | 18.20 | 18.25 |

^1^ Per kilogram of mineral premix (g/kg): MnSO_4_·H_2_O (31.8% Mn), 0.86 g； MgSO_4_·H_2_O（15.0% Mg）, 133.33g; FeSO_4_·H_2_O (30.0% Fe）, 9.29g; ZnSO_4_·H_2_O (34.5% Zn), 2.48 g; CuSO_4_·5H_2_O (25.1% Cu), 0.62 g; Ca(IO_3_)_2_（3.2% I）,1.72g; Na_2_SeO_3_（0.45%），2.22 g; with corn starch filled to 1 kg.

^2^ Per kilogram of vitamin premix (g/kg): Vitamin A acetate (500 000 IU/g), 0.40 g; Vitamin D3 (500 000 IU/g), 0.25g; DL-α tocopherol acetate (47.5%), 7.63 g; Vitamin K3 (50%), 2.00 g; Vitamin B12 (1%), 0.20 g; D-biotin (2%), 0.75 g; Folic acid (95%), 0.16 g; Thiamine nitrate (98%), 0.13 g; Vitamin C (95%), 12.05 g; Niacinamide (99%), 1.41 g; Inositol (96.5%), 31.08 g; Calcium D-pantothenate (93.1%), 1.76 g; Riboflavin (80%), 1.13 g; Pyridoxine hydrochloride (97.5%), 0.38 g; with corn starch filled to 1 kg.

^3^ The choline chloride premix contains 266.48 g of choline chloride (50%) per kilogram, the rest diluted with corn starch to 1 kg.

^4^Crude protein, crude lipid, and energy contents were measured values.

**Table S2**

The different peptides of the enzymatic cottonseed protein (ECP) as a proportion of total protein.

| Molecular mass | Proportion, % |
| --- | --- |
| ＞10000 Da | 36.0 |
| 3000-10000 Da | 10.0 |
| 1000-3000 Da | 16.0 |
| 150-1000 Da | 34.0 |
| ＜150 Da | 5.0 |

**Table S3**

The primer sequences and accession numbers of genes were selected for analysis by real-time PCR.

| Target gene | Forward (5’→3’) | Reverse (5’→3’) | Accession number |
| --- | --- | --- | --- |
| *tnfα* | ATCAGGTGAACGCTGATGCT | GTGTTGAGGGAAGGGGTCTG | [XM_027165847.2](https://www.ncbi.nlm.nih.gov/nuccore/XM_027165847.2) |
| *il1β* | TAGGCATAGAGGAGGTAA | AAGGTGTTCAGGGAGTCA | Chen et al [1] |
| *tgfβ* | ATCTTCTCGCTGTCCACTTGC | CGGTCTCGGTGTTGTCCTG | [XM_047818049.1](https://www.ncbi.nlm.nih.gov/entrez/viewer.fcgi?db=nucleotide&id=2227670969) |
| *il10* | CTCCTCCCCCTGAGGATTCA | CGGATCACGGCGTATGAAGA | [XM_027144360.2](https://www.ncbi.nlm.nih.gov/nuccore/XM_027144360.2) |
| *jak1* | GGACTATTTGCCCCGACACA | TCTGAGAGCCCAGGTACTCC | [XM_027153705.2](https://www.ncbi.nlm.nih.gov/entrez/viewer.fcgi?db=nucleotide&id=2227544091) |
| *iκb* | TTTCGGAGGAGATGGAGAGA | CTGTTCAGGTACGGGTCGTT | Cheng et al [2] |
| *iκκs* | GAGGCTTGGAACAGGAGGATTTGG | CTCAAGGCGACAGAGCTTCACAG | Cheng et al [2] |
| *ap1* | GTTACGTGGAGCTGCTGGAT | CAGGTCAGCCTGTTCGATGG | XM_027144952.2 |
| *il6* | ATGCCTCACCTAGAGCAGGA | GTGAAGCTGTGCAGAATGGA | XM_027144952 |
| *socs1a* | GATGTTTCTATAATCCGTTTGC | AGCTACAGGGCTCCTTCG | [XM_027156836.2](https://www.ncbi.nlm.nih.gov/nuccore/XM_027156836.2) |
| *socs3b* | GCTCCCGTTCCAGCATAG | GGTCAGGCACAGCAAGTT | [XM_027140622.2](https://www.ncbi.nlm.nih.gov/nuccore/XM_027140622.2) |
| *cox2* | CCTGAGCTGTTCCCGCATTA | GAATACTCCTGGGCGGGAAG | NC_015888.1 |
| *jnk* | ACGTTATCAGCACCTGAGGC | TGACATTCTGGAAGGGCCTG | [XM_027167562.2](https://www.ncbi.nlm.nih.gov/nuccore/XM_027167562.2) |
| *erk* | GGCTGTTCCCAAATGCTGAC | AACTTGAATGGTGCTTCGGC | [NM_002745.5](https://www.ncbi.nlm.nih.gov/nuccore/NM_002745.5) |

1.Chen H, Yuan G, Su J, Liu X. Hematological and immune genes responses in yellow catfish (*Pelteobagrus fulvidraco*) with septicemia induced by Edwardsiella ictaluri. Fish Shellfish Immunol. 2020, 97: 531-539.<http://dx.doi.org/10.1016/j.fsi.2019.11.071>

2.Cheng K, Tang Q, Guo X, Karow N A, Wang C. High dose of dietary vitamin D3 modulated the yellow catfish (*Pelteobagrus fulvidraco*) splenic innate immune response after Edwardsiella ictaluri infection. Fish Shellfish Immunol. 2020, 100: 41-48 [http://dx.doi.org/ [10.1016/j.fsi.2020.03.005](https://www.x-mol.com/paperRedirect/1234993868583038976)](http://dx.doi.org/10.1016/j.fsi.2019.11.071)

**Table S4**

Target proteins, dilution factor, antibody cat. no. and antibody source of proteins selected for analysis by western blotting.

| Target proteins | Dilution ratio | Antibody cat. no. | antibody source |
| --- | --- | --- | --- |
| p-STAT3 | 1:1000 | ET1603-40 | Huabio, China |
| STAT3 | 1:1000 | ET1607-38 | Huabio, China |
| p-STAT1 | 1:1000 | HA722083 | Huabio, China |
| STAT1 | 1:3000 | ET1612-22 | Huabio, China |
| F4/80 | 1:100 | IRS052 | Huabio, China |
| NF-κB | 1:60000 | A22331 | ABclonal, China |
| p-NF-κB | 1:10000 | AP0124 | ABclonal, China |
| NLRP3 | 1:5000 | A21906 | ABclonal, China |
| p-RIP1 | 1:1000 | AP1230 | ABclonal, China |
| p-RIP3 | 1:100 | AP1257 | ABclonal, China |
| p-MLKL | 1:100 | AP1244 | ABclonal, China |
| p-P38/MAPK | 1:1000 | ER1903-01 | Huabio, China |
| P38/MAPK | 1:1000 | ET1602-26 | Huabio, China |
| GAPDH | 1:1000 | ET1601-4 | Huabio, China |
