# Supplementary figures and images for: Enzymatic cottonseed protein alleviates DSS-induced enteritis in juvenile yellow catfish (Pelteobagrus fulvidraco): focus on macrophage polarization and necroptosis in the intestine
Source: J Anim Sci Biotechnol. 2025 Aug 26;16:119. doi: 10.1186/s40104-025-01248-z (PMC12379407; doi:10.1186/s40104-025-01248-z)

## GAPDF:

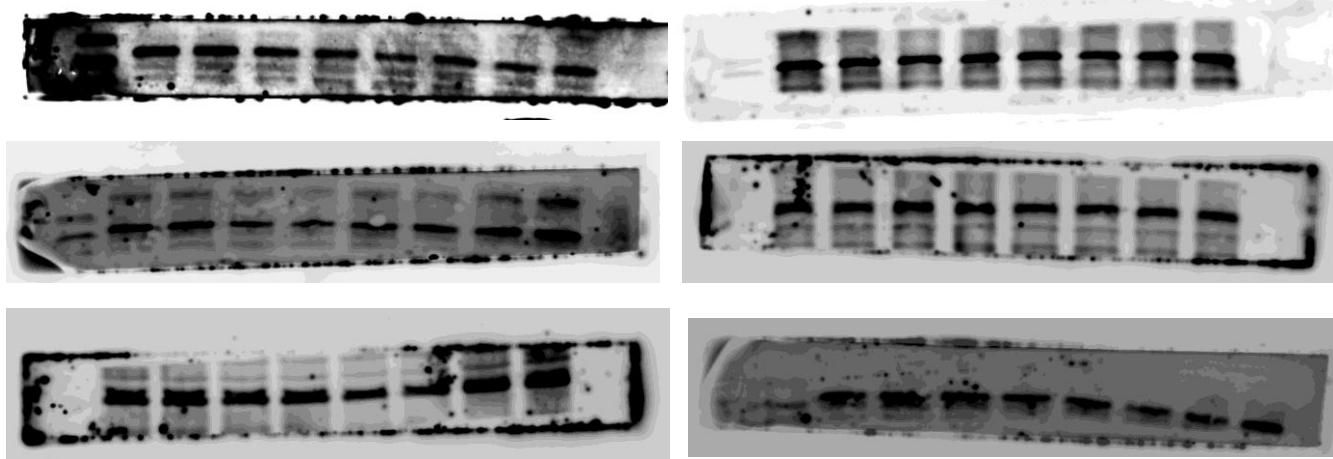

**p-STAT1**

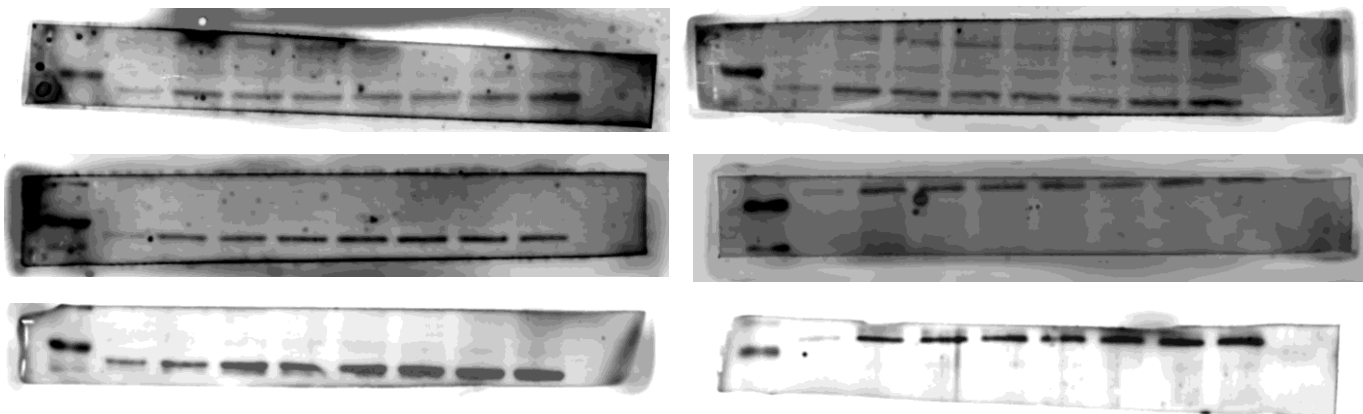

# STAT1

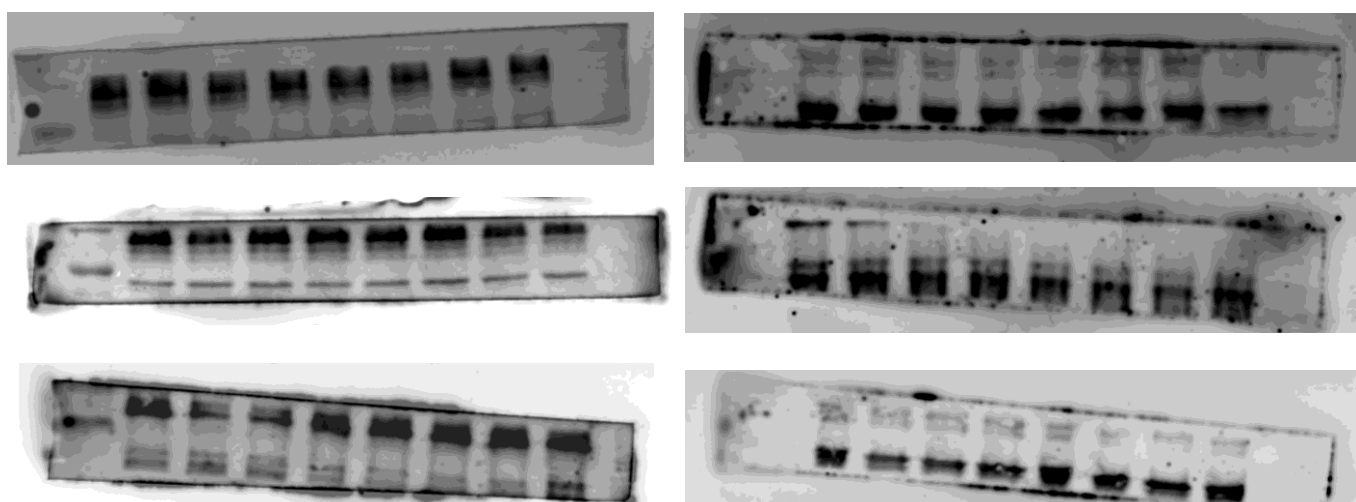

p-STAT3

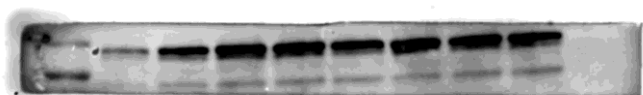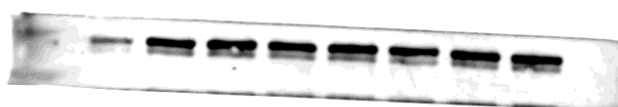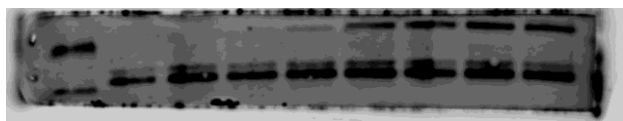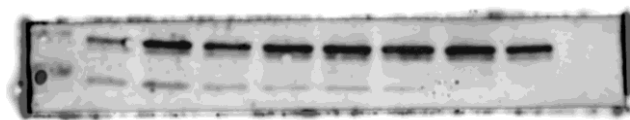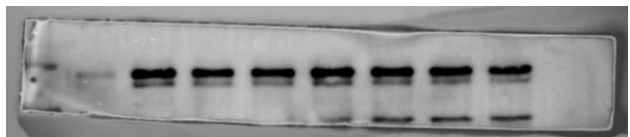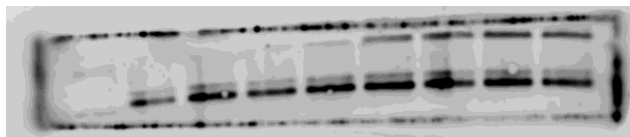

STAT3

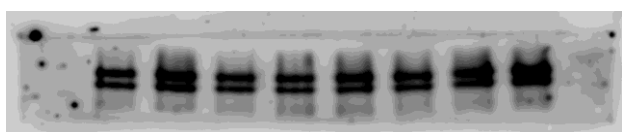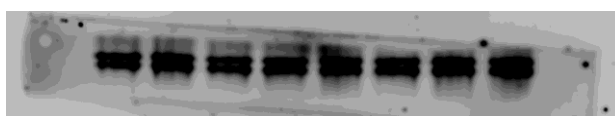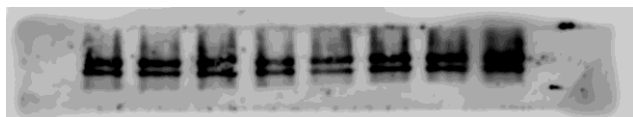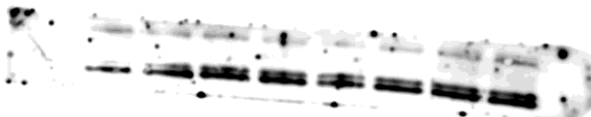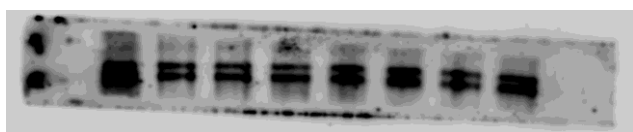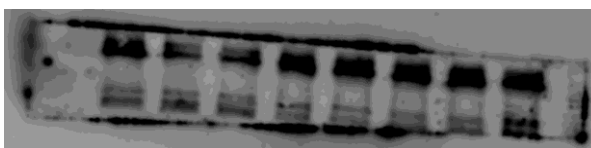

p-RIP1

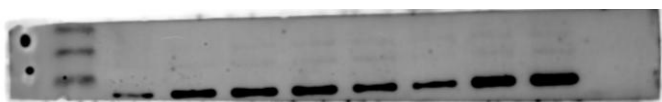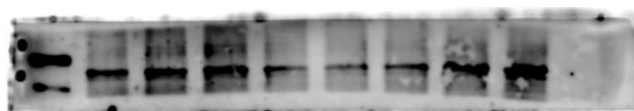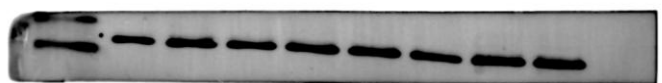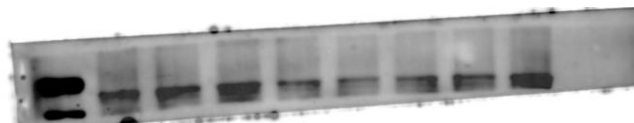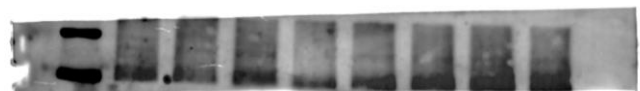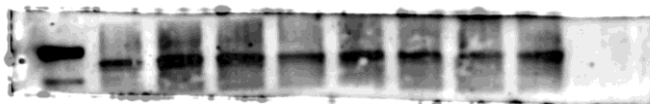

## NLRP3

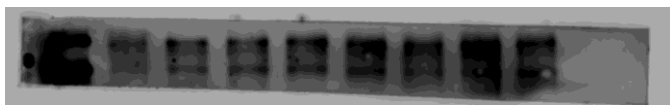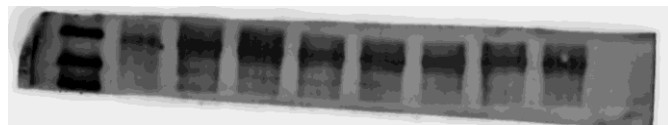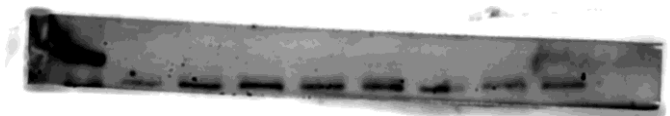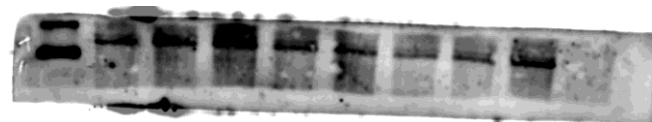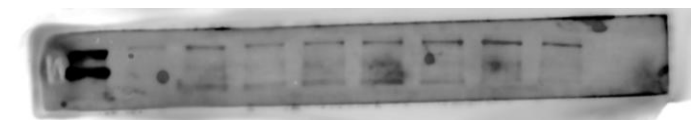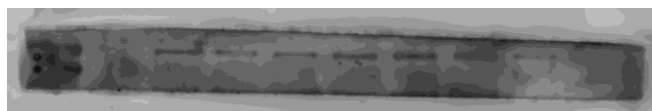

## p-NF- $\kappa$ B

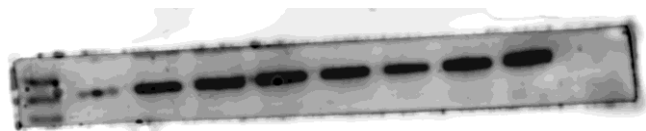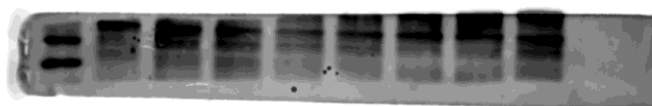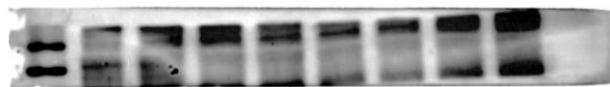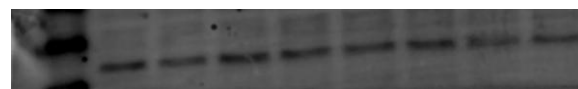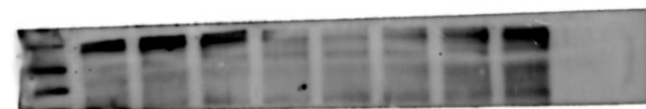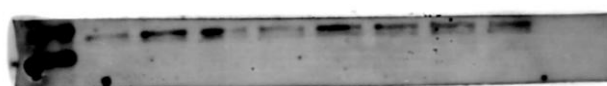

## NF- $\kappa$ B

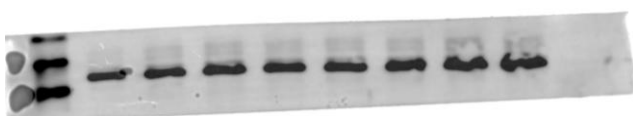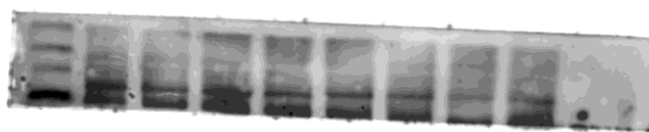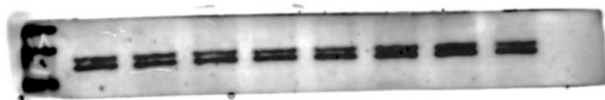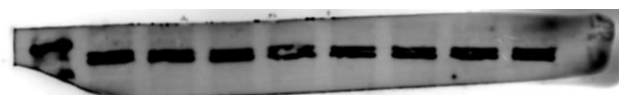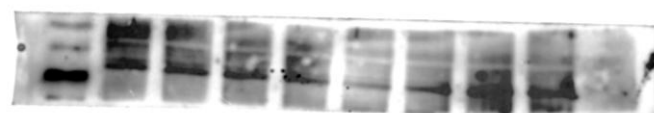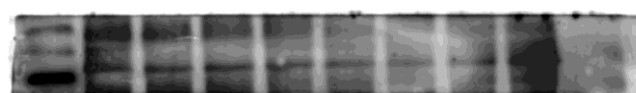

**p-p38 MAPK**

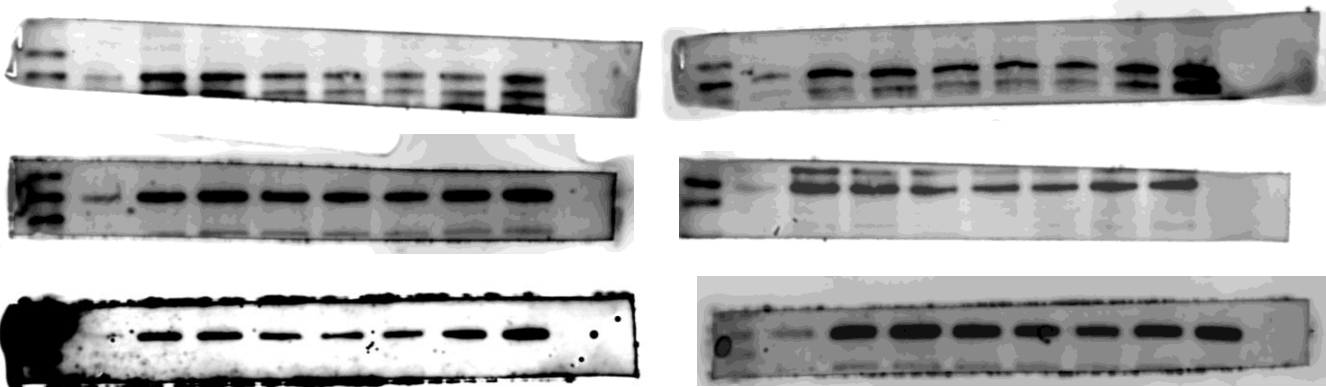

**p38 MAPK**

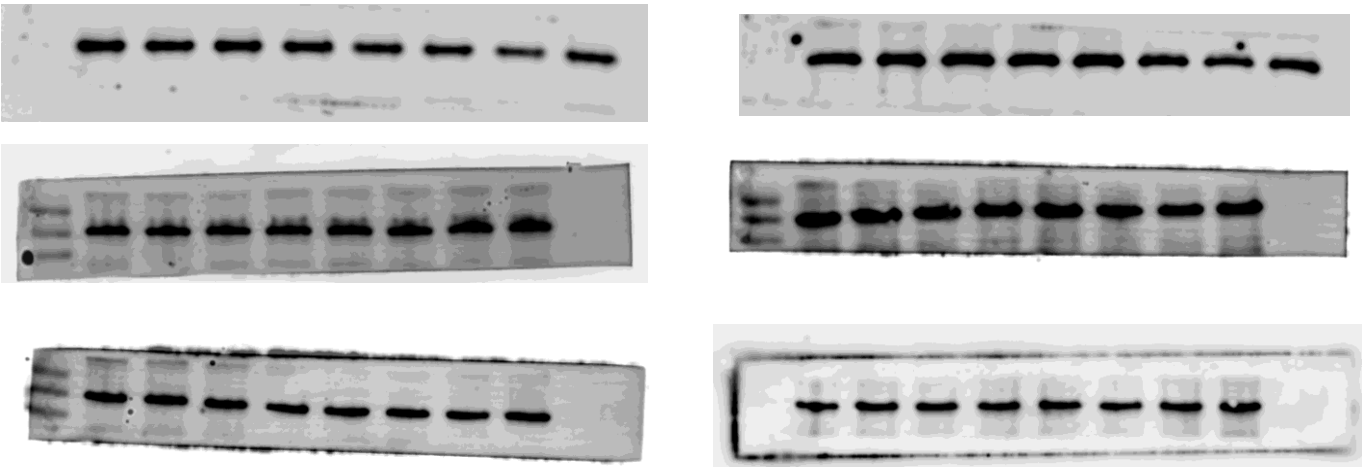

Supplement: Supplementary file 2 — Supplementary Material 2: Original Western blot images for all protein bands analyzed in the study. [file 40104_2025_1248_MOESM2_ESM.pdf]
